# Interface mobility between monomers in dimeric bovine ATP synthase participates in the ultrastructure of inner mitochondrial membranes

**DOI:** 10.1073/pnas.2021012118

**Published:** 2021-02-04

**Authors:** Tobias E. Spikes, Martin G. Montgomery, John E. Walker

**Affiliations:** ^a^The Medical Research Council Mitochondrial Biology Unit, University of Cambridge, Cambridge, CB2 0XY, United Kingdom

**Keywords:** bovine mitochondria, ATP synthase, dimer, monomer-monomer interface, mobility

## Abstract

The ATP synthase complexes in mitochondria make the ATP required to sustain life by a rotary mechanism. Their membrane domains are embedded in the inner membranes of the organelle, and they dimerize via interactions between their membrane domains. The dimers form extensive chains along the tips of the cristae with the two rows of monomeric catalytic domains extending into the mitochondrial matrix at an angle to each other. Disruption of the interface between dimers by mutation affects the morphology of the cristae severely. By analysis of particles of purified dimeric bovine ATP synthase by cryo-electron microscopy, we have shown that the angle between the central rotatory axes of the monomeric complexes varies between ca. 76 and 95°. These particles represent active dimeric ATP synthase. Some angular variations arise directly from the catalytic mechanism of the enzyme, and others are independent of catalysis. The monomer–monomer interaction is mediated mainly by j subunits attached to the surface of wedge-shaped protein-lipid structures in the membrane domain of the complex, and the angular variation arises from rotational and translational changes in this interaction, and combinations of both. The structures also suggest how the dimeric ATP synthases might be interacting with each other to form the characteristic rows along the tips of the cristae via other interwedge contacts, molding themselves to the range of oligomeric arrangements observed by tomography of mitochondrial membranes, and at the same time allowing the ATP synthase to operate under the range of physiological conditions that influence the structure of the cristae.

## Significance

Mitochondria are the powerhouses of eukaryotic cells. Pairs of molecular machines with a rotary action, called ATP synthase, are embedded in their inner membranes and produce adenosine triphosphate, ATP, the fuel of life. These dimers form long rows on cristae tips, helping to endow them with their characteristic and mobile tubular shape. Our structural analyses of bovine dimers show that some structural changes depend on catalysis and others are independent. The monomers pivot and translate at wedge-shaped structures in their membrane domains. The structures suggest how dimeric ATP synthases might interact and fashion themselves to the range of oligomeric arrangements observed in mitochondria, whilst allowing the ATP synthase to produce ATP under a wide range of physiological conditions.

The ATP synthase complexes embedded in the inner membranes of mitochondria (IMM) are rotary machines that make the ATP required to sustain life (1). Energy from respiration generates a proton motive force (pmf) across the IMM, and this pmf is applied directly to the membrane embedded sector of the rotor of the ATP synthase via a Grotthuss chain of water molecules, thereby generating rotational torque to turn the rotor in the synthetic direction of rotation (2). In mammals, this membrane sector of the rotor is composed of a ring of eight c subunits and is attached to an extramembranous central stalk assembly of subunits γ, δ and ε, which penetrates into the extramembranous α_3_β_3_-catalytic domain ca. 90 Å distant from the membrane domain (2, 3). Three of the six interfaces between α- and β-subunits contain the catalytic sites of the enzyme, and as the rotor turns in 120° steps during the rotary cycle, these sites undergo a series of structural changes that are associated in turn with the binding of substrates, and the formation and release of ATP from each site (4). The catalytic domain is also attached to the membrane domain via a peripheral stalk (PS), an elongated rod that reaches ca. 150 Å between the catalytic domain and the surface of the IMM (2, 5, 6). The core of the rod is provided by a long α-helix, bH3, which extends between the α_3_β_3_-catalytic domain and the IMM, and then penetrates and crosses the IMM to reach the intermembrane space (IMS) and then returns to the mitochondrial matrix via a second transmembrane span. The membrane extrinsic part of bH3 is supported and largely rigidified by other α-helices in the d and F_6_ subunits and in the membrane extrinsic region of subunit ATP8, all bound approximately parallel to the membrane extrinsic region of bH3. The upper part of the rod is attached via the C-terminal α-helix of subunit b, bH4, to the C-terminal domain of the OSCP subunit, and the N-terminal domain of this subunit binds to the N-terminal regions of the three α-subunits. The two domains of the OSCP are joined by a flexible linker, which provides the PS with a universal joint. During the rotary catalytic cycle, the α_3_β_3_-domain rocks from side to side, as noted before (7–10), with little or no displacement perpendicular to the lateral motion of the PS toward the central axis of the rotor (2), and the PS accommodates this rocking motion via the combined action of the universal joint and a hinge in the PS close to the surface of the IMM (2). The membrane bound N-terminal region of the b subunit is folded into two further α-helices, bH1 and bH2, and together with the transmembrane domain of bH3 they form the skeleton of a wedge-shaped structure in the membrane domain (2), with bH1 sitting on top on the matrix side of the IMM and transmembrane α-helices bH2 and bH3 subtending an angle of ca. 45°. Supernumerary subunits e, f, and g also contribute to the structure of the wedge. The transmembrane α-helices eH1 and gH3 of subunits e and g augment α-helix bH2, and the top of the wedge on the matrix side of the membrane is provided by four amphipathic α-helices, two in each of the g and f subunits, lying in the lipid head-group region. Internal cavities in the wedge are occupied by five specifically bound lipids, three cardiolipins (CDL1, CDL2, and CDL3), and two less well-defined phospholipids modeled tentatively as phosphatidyl glycerols (LHG4 and LHG5). These phospholipids enhance the stability of the wedge (2). Subunit j is associated with the wedge, and in the dimeric complex, the two j subunits interact with each other across the monomer-monomer interface. In the IMM, the dimeric ATP synthase complexes occupy the tips of the cristae, and the dimers are arranged in long rows along the tips of the cristae (11–13). How the dimers are held together in higher oligomers is uncertain, but protein–protein interactions in the membrane domains of dimers may provide interdimer tethers (2, 14). If the capacity of the monomeric complexes to form dimers is removed, for example by deletion of one of the wedge components, the structure of the IMM changes dramatically, and the cristae tubes disappear (15–21). Thus, the dimerization of the ATP synthase appears to be a determinant of the formation of cristae. These dynamic ATP synthase complexes operate in the ever-changing structural context of the mitochondria themselves. The organelles are constantly being remodeled by fission of mitochondrial networks and by fusion of vesicular mitochondria, influenced, for example, by cellular conditions such as energetic state, the cell cycle, or by proapoptotic or cell-death factors (22). The fusion and fission events involve changes in both inner and outer membranes, in the latter case again influencing the structures of the cristae and the disposition of the dimeric ATP synthase complexes within them. During these dynamic events and under conditions of active cellular growth, the IMM needs to remain coupled to ATP synthesis. Therefore, in order to remain active, the dimeric ATP synthase not only has to accommodate changes in the monomer-monomer interface that arise directly from its own catalytic activity, but also others that stem from the dynamics of the organelle itself, and without dissipating the pmf by leakage of protons through the monomer-monomer interface. In tomographic reconstructions of mitochondrial cristae, zig-zagging, lateral bending, perpendicular curvature, and nonuniform packing of rows of dimers of ATP synthase have been observed (12, 17, 23), illustrating the wide range of varying modes of oligomerization under which the ATP synthase complexes operate.

As shown here, the interaction between the two wedges linking the monomers in the dimeric ATP synthase is not a constant feature. It changes to accommodate both the rocking motions of the membrane extrinsic catalytic domain associated with catalysis and other motions that are independent of catalysis observed in the isolated dimeric ATP synthase. These pivoting motions and sliding translations between the monomeric complexes are intrinsic features of the dimeric ATP synthase. They allow it to operate in the ever-changing ultrastructure of the IMM and also to contribute to their ultrastructure.

## Results and Discussion

### Classification of Dimeric Particles of Bovine ATP Synthase.

As described before, three datasets of 4,267, 2,238, and 4,096 dose-fractionated exposures of the purified dimeric bovine ATP synthase were collected, and structures of the monomeric enzyme were produced by single-particle analysis of 176,710 particle coordinates (2). Although each monomer is inhibited by a monomeric form of the inhibitor protein, IF_1_, the inhibitor has trapped dimeric states where each monomeric catalytic domain contains the same three rotational points in the active catalytic cycle. The positions of these three rotational points relative to the peripheral stalk give rise to three states of the intact monomeric ATP synthase named s1, s2, and s3. Complete structures of the monomeric complex were built (2), and by combining states s1, s2, and s3 (*SI Appendix*, Table S1), models of dimeric complexes were constructed in a range of combinations of catalytic states and substates (*SI Appendix*, Scheme S1 and Table S2). The substates arise from changes that are independent of catalysis in the relative disposition of the two monomers to each other (*SI Appendix*, Scheme S2). By hierarchical classification of the earlier dataset (*SI Appendix*, Scheme S1) and subsequent refinement, 154,130 particles were resolved into nine discrete classes of the dimeric assembly representing rotational states [s1:s1], [s1:s2], [s1:s3], [s2:s1], [s2:s2], [s2:s3], [s3:s1], [s3:s2], and [s3:s3] at resolutions of 9.2, 11.9, 9.0, 9.4, 8.5, 10.7, 9.7, 11.4, and 13.1 Å, respectively. Composite dimer models were created by rigid body fitting of the maps and associated atomic coordinates (2) into these lower resolution envelopes. Further classification of the particles in each catalytic state (*SI Appendix*, Scheme S2) revealed 59 additional substates of the dimer, at resolutions ranging from 13.8 to 23.8 Å. The substates are distinguished from discrete classes of the dimeric assembly in defined rotational states by sequential lettering and are grouped according to catalytic state. For example, the first, second, and third substates of the ATP synthase dimer in which the right and left monomers are in catalytic states 1 and 3, respectively, are referred to as dimer substates [s1:s3a], [s1:s3b], and [s1:s3c].

### Changes in the Monomer-Monomer Interface during Catalysis.

**Fig. 1.**
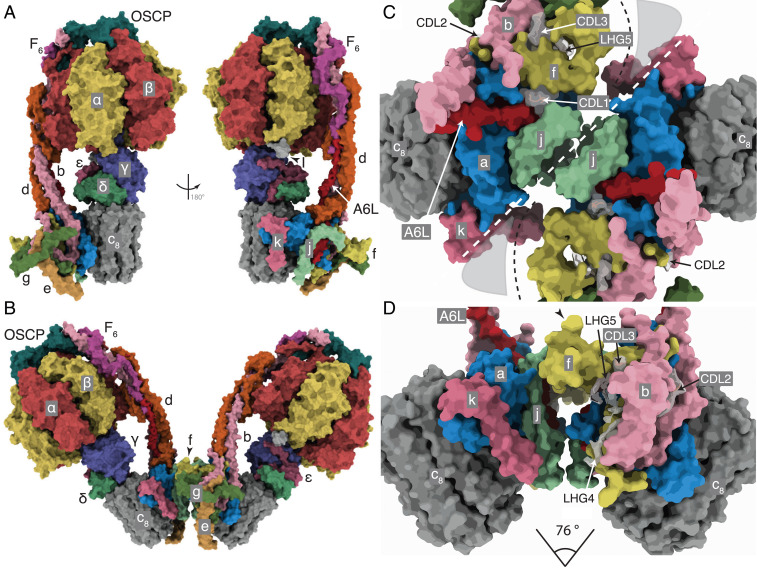
The structure of the bovine ATP synthase and the monomer-monomer interfaces in the membrane domains of dimers. (*A* and *B*) The structures and subunit compositions of the bovine ATP synthase monomer in rotational state 3 (Protein Data Bank [PDB] ID code 6ZQN (2)) and the dimer in state [s1:s1] (PDB ID code 7AJB), respectively, shown as solvent excluded molecular surfaces. The s1:s1 dimer was created by docking two s1 monomers (PDB ID code 6ZPO) into the lower resolution s1:s1 dimer map. The α, β, γ, δ, and ε subunits of the F_1_ catalytic domain are red, yellow, blue, indigo, and green, respectively, with the central stalk (subunits γ, δ, and ε) attached to the c_8_ ring (dark gray) in the membrane domain in contact with subunit a or ATP6 (cornflower blue). The PS subunits OSCP, b, d, and F_6_ are teal, light pink, orange, and magenta, respectively, and the A6L subunit is brick red. In the region of the monomer-monomer interface, subunits e, f, g, j, and k are khaki, straw yellow, forest green, sea-foam green, and dark pink, respectively. Cardiolipin (CDL) and phosphatidyl-glycerol (LHG) are transparent gray. In *A*, the monomeric complex is viewed in two rotated positions to reveal the positions of all subunits, with the rotatory axes aligned vertically. I denotes residues 1–60 of the inhibitor protein IF_1_. In *B*, the dimer is viewed from within the plane of the IMM. (*C* and *D*) The solvent excluded molecular surfaces of subunits in the membrane domains of the dimeric complexes viewed between the two peripheral stalks. (*C*) The complex is viewed from the matrix side of the IMM with the monomer:monomer interfaces indicated by the white dashed line. The black dashed line denotes the protein boundary between the monomers, adjacent to a region occupied by nonspecific lipids and the detergent micelle (gray shading). (*D*) The orthologous view in the plane of the IMM with the monomer:monomer interfaces exposed by removal of subunits e, g, and d. The angles between the axis of rotation in each monomer indicated beneath were estimated by calculating two centroids for residues 2 and 38 in each bovine c_8_ ring. The axis connecting the two centroids approximates to the rotatory axis of the c ring. The centroids and connecting axes were calculated with the Structure Measurements tool-set in Chimera (35). The angle of intersection was measured from the models aligned with these axes orthogonal to the direction of the view.

**Fig. 2.**
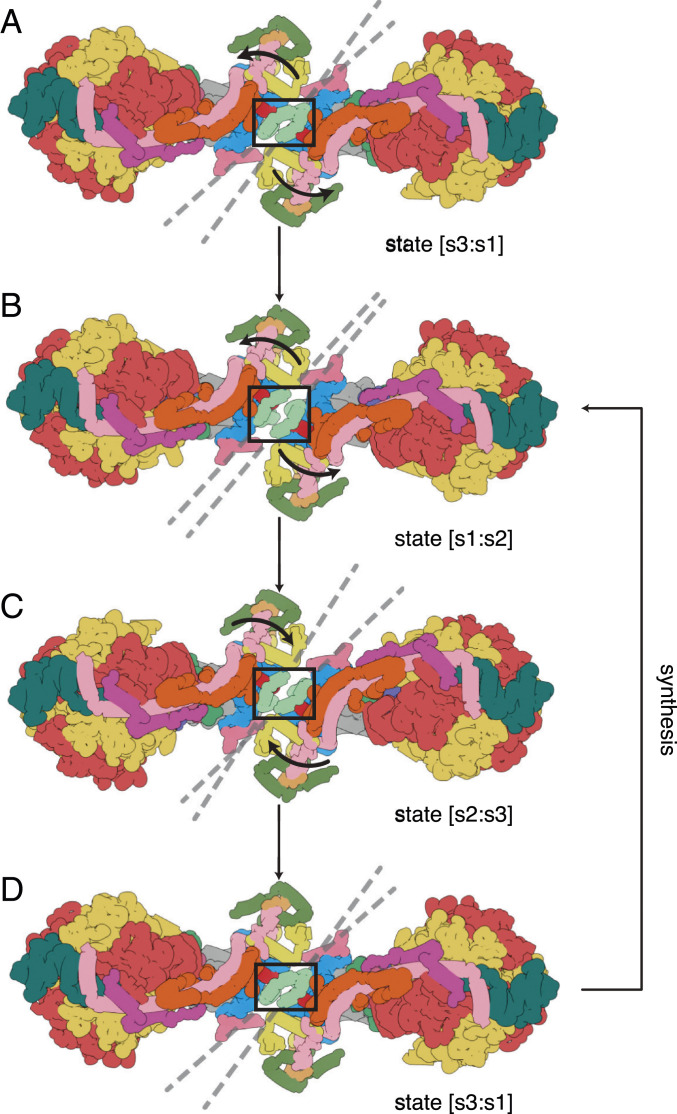
Pivoting of the membrane domains of bovine ATP synthase about the matrix contact between j subunits during ATP synthesis. Subunit j is sea-foam green inside the black box. For colors of other subunits, see Fig. 1 legend. (*A*) The state [s3:s1] dimer. Dashed lines indicate the central axis of amphipathic α-helices jH1 (residues 1–19), which lie in the plane of the matrix leaflet of the membrane. During the synthetic rotary cycle, each monomer progresses from state 1 to state 2 to state 3 and so forth. (*B*) Rotation of the membrane domain about the contact point in jH1 during the transition from state [s3:s1] to state [s1:s2], with accompanying displacement of subunits e, f, and g, and the transmembrane α-helices bH2 (residues 33–47) and bH3 (residues 55–73), moving the wedge outwards or inwards as indicated by the arrows. The membrane extrinsic F_1_ domains remain approximately stationary, and the rocking motion associated with the asymmetry of the central stalk is transmitted to the membrane domain via the universal joint between N- and C-terminal domains of the OSCP in the PS (2). (*C*) A similar pivoting motion occurs in the transition from state [s1:s2] to state [s2:s3]. (*D*) Completion of the rotary cycle by the transition from state [s3:s1] to state [s1:s2]. The synthetic rotary cycle continues via *B* as indicated by the arrow on the right. The scheme was constructed with composite atomic models of monomeric bovine ATP synthase, which were rigid body fitted into the consensus dimer structures produced according to *SI Appendix*, Scheme S1. This figure relates to Movie S3. In both this figure and Movie S3, side chains have been removed and secondary structure elements have been dilated to produce the diagrammatic nature of the figure, which is inferred from lower resolution structures of the intact dimeric complex.

**Fig. 3.**
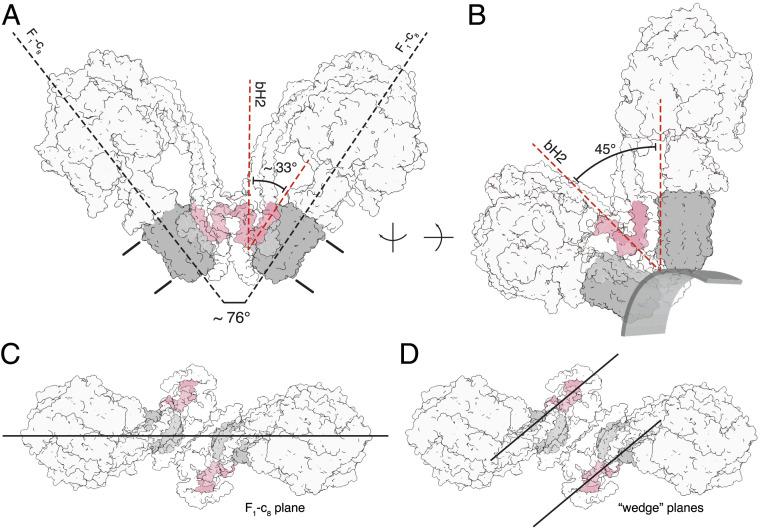
Relationship between the inclined membrane wedge in the monomeric membrane domain and the association of two monomers into a dimer. The dimer in state [s2:s2] is shown. The c_8_ ring is gray, and transmembrane α-helices bH2 and bH3 of subunit b (pink) define the wedge angle (2). The rest of the structure is shown in silhouette. (*A*) Side view showing the relationship between the angles of rotatory axes and the wedge angles when measured from the same reference point, with both rotatory axes aligned in plane. The angle between bH2 and bH3 in this view is 33°, approximately half of the dimer angle. The approximate boundary of the membrane is indicated; in *B,* the rotatory axis is aligned vertically and the structure is rotated so that bH2 and bH3 in the wedge of the right-hand monomer are in the plane of the paper. The 45° wedge angle is a measure of the inclination of the transmembrane α-helix bH2 relative to bH3 and the rotatory axis of the enzyme, as required for the wedge to provide the membrane curvature in the membrane domain of each monomer. The approximate boundary of the lower leaflet is indicated to provide perspective. (*C* and *D*) Top views showing the differences between the angle measurement planes to explain why a simple doubling of the wedge angle does not produce the rotatory axis angle. In the context of the angle variations observed in the consensus reconstructions, the wedge angle and dimer, or rotatory axis, angle are separate considerations. The wedge angle does not change, rather, the way in which the membrane domains are arranged relative to one another does, as described in Fig. 2 and Movie S3.

In the dimeric complex, the monomer-monomer interface in the membrane domain consists of contacts between the two j subunits each on the external surface of the wedge (Fig. 1 and *SI Appendix*, Fig. S1*A*). From residues 1–20, subunit j is folded into an amphipathic α-helix jH1, which lies in the lipid head group region on the matrix side of the IMM where it interacts with the C-terminal region of transmembrane α-helix A6LH1 and CDL1. The negatively charged headgroup of CDL1 is bound to jK8, and to residues fQ38 and fY42 of the f subunit in the wedge and residues aT33, and A6LK27 and A6LK30 (*SI Appendix*, Fig. S1*B*). Transmembrane α-helix jH2 (with residues 22–39 forming the transmembrane span) lies adjacent to A6LH1 on the opposite side of aH1 to the wedge and is associated with subunit a via interactions between its N-terminal region and residues 107–108, 110–111, and 114–115 of aH4 (*SI Appendix*, Fig. S1 *A* and *B*). The C-terminal region of jH2 (residues 40–49) protrudes into the IMS. Residues j50–60 were not modeled as they were not well-defined in the cryo-electron microscopy reconstructions. They are predicted to have an extended structure (*SI Appendix*, Fig. S2) that interacts with the same region in the adjacent j subunit, as indicated by the structures of the dimers in various rotational states and many of their substates, where density beyond the modeled residues can be observed (*SI Appendix*, Fig. S1 *D*–*F*). However, the interactions between the two j subunits are mobile, and the observed angle between the central axes of the central stalk or the PS of the monomers ranges from ca. 75 to 86° in the nine rotational catalytic states (Movies S1 and S2). This range of angles arises by the rigid body of the wedge and the rest of the membrane domain pivoting about contact points between the amphipathic α-helices jH1 (residues 1–20), provided by residues 3, 7, 10, 11, and 14 (Figs. 2 and 3 and *SI Appendix*, Fig. S1). The two jH1 α-helices are oriented in the plane of the IMM on the matrix side as the enzyme contorts during the rotational cycle (Figs. 1 and 2 and Movie S3). Residues 3, 7, 10, 11, and 14 in jH1 project toward the interface, possibly interacting via an intervening lipid. This pivoting reduces the net side-to-side displacement of the catalytic domain arising from the rotation of the asymmetrical central stalk and accommodates other changes in the structure of the dynamic mitochondrial cristae.

### Changes in the Monomer-Monomer Interface Unrelated to Catalysis.

**Fig. 4.**
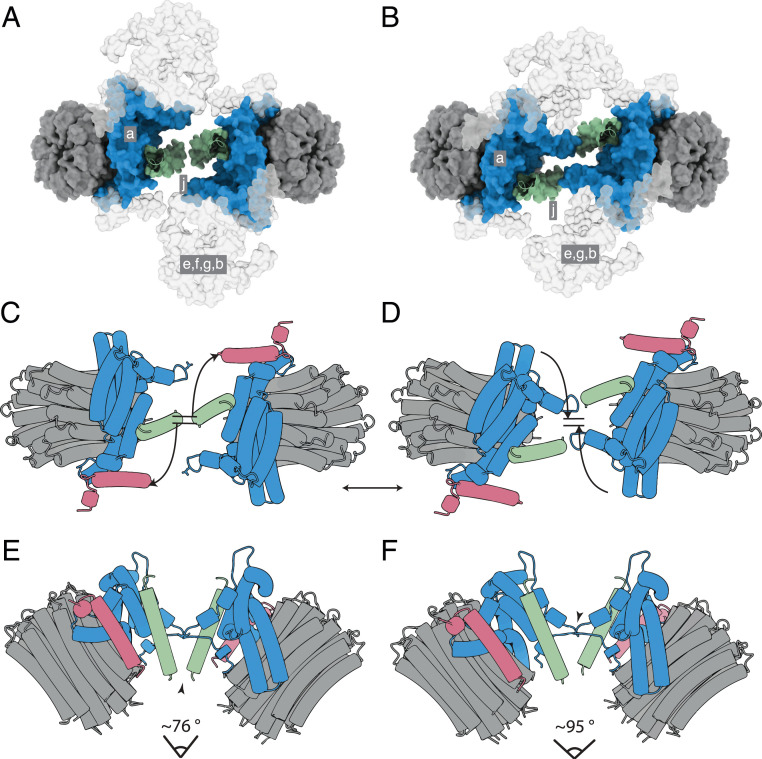
Dispositions of subunits a and j in the monomer-monomer interface of dimeric bovine ATP synthase at narrowest and widest extremities of angles between rotatory axes. Subunits a, j, and k are blue, green, and pink, respectively, and the c_8_ rings are gray. (*A* and *B*) Views from the matrix side of the IMM of the monomer-monomer interfaces in the membrane domains of bovine dimers with angles between rotatory axes of ca. 76° and ca. 95°, respectively. In *A* and *B*, respectively, the dimeric substate [s2:s2c] (*SI Appendix*, Schemes S1 and S2) and the dimeric substate [s2:s2b] (*SI Appendix*, Scheme S2) are shown as examples. The positions of subunits e, f, g, and the membrane domain of subunit b are indicated in gray silhouette. For clarity, the F_1_ domains and all PS subunits have been removed. (*C*–*F*) Schematic representation of the monomer-monomer interfaces, viewed in *C* and *D* as in *A* and *B*, respectively, and in *E* and *F* orthogonal to the plane of the membrane. In *E*, the arrowhead indicates an interaction between the C-terminal residues of the two j subunits. In *F*, the arrowhead indicates a possible interaction of residues 12–19 in a loop proceeding αH1 of the a subunit. In *C* and *D*, the curved arrows indicate the approximate compound rotation of the membrane domains that occurs as the angle of the rotatory axis changes between the dimer substates. This figure relates to Movies S5 and S6.

Other variations in motion occurring within groups of particles of a defined catalytic state are substates that are independent of catalysis. They fall into two categories, namely those where the angle between rotatory axes is less than 90° (Movie S4) and others where the angle is greater than 90° (Movies S5 and S6). The motions in the former category are similar to those that occur during catalysis in that the relative dispositions of the membrane domains change by a pivoting motion about the interface between j subunits, with the C-terminal IMS protrusions remaining in contact. The flexibility of this pivot is an intrinsic property of the interface, and it has other roles beyond that described above of dissipating the rocking of the catalytic domain in the mitochondrial cristae. Most notably, it provides a mechanism to accommodate the general fluidity of the membrane in the region of curvature associated with the dimeric ATP synthases and the rearrangements that occur. The second category of motions between substates, where the angle between rotatory axes exceeds 90°, arises from sliding and twisting along the monomer-monomer interface presented by the a and j subunits and the membrane wedge, thereby effecting a compound rotation of one monomer relative to the other (Fig. 4 and Movies S4 and S5). For example, in Fig. 4, where the structures of the ATP synthase dimers in the substates [s2:s2c] and [s2:s2b] are compared, the positions of the protrusion of subunit j into the IMS and of the loop region (residues 12–19) in subunit a differ significantly in the two structures (Movies S5 and S6). In substate [s2:s2c], the C-terminal residues of subunit j are in contact in the IMS as in Fig. 4*C*. In contrast, in substate [s2:s2b], these residues are displaced by ca. 20 Å and the two loops consisting of residues 12–19 of subunit a appear to make contact with each other, as in Fig. 4*D*. This compound rigid body rotation of the monomers changes the angle between the rotatory axes significantly and also rearranges the amphipathic α-helices lying in the IMS leaflet of the membrane (Fig. 4 and Movies S5 and S6). In the IMM, this transformation could be part of a mechanism for the dimers and oligomeric rows to adapt to the convolutions of the cristae, with the two contact positions representing a simple steric mechanism that restrains the rotation to upper and lower limits. In this mechanism, the wide angle conformation (as observed in substate [s2:s2b]; see Fig. 4 *B*, *D*, and *F*) rests at the position where the two a subunits are in contact and is prevented from widening further by aH1, and the conformation with a shallow angle (for example, the substate [s2:s2c] in Fig. 4 *A*, *C*, and *E*) rests at the position where the two j subunits are in contact in the IMS. There is probably a continuum of substates between the two extremes. The conformers with shallow angles between rotatory axes of ca. 76° to 86° were the most abundant observed substates (*SI Appendix*, Fig. S3 and Scheme S2). The highly dynamic nature of the dimeric complex is demonstrated by the compilation of all the observed conformers in Movie S7.

It cannot be excluded that the wide angle substates with rotatory axis angles greater than ca. 95°, which depart significantly from the consensus structures with rotatory axis angles of ca. 76–86°, arise from the influence of the detergent micelle and the lack of the interactions between dimers that occur in native membranes. In curved membranes, an estimated tension of ca. 10 mN/m is exerted on mechanosensitive channels (24, 25), and it is possible that force from mitochondrial lipids at the cristae apices stabilizes the dimer interface, and in situ this lateral force might reduce the range of motion observed in isolated monomers. The subtomographic reconstructions of dimeric ATP synthases from mammalian mitochondria, where rotatory axis angles of ca. 80° have been reported, represent the average structures of many individual molecules, or subtomograms, within the data (12), similar to the consensus dimer in *SI Appendix*, Scheme S1. Therefore, they do not exclude the possibility of large variations in the angle of the rotatory axis, especially if those conformations arise rarely, for example at cristae ultrastructures with sharp negative curvature, or during dynamic rearrangements of the cristae, and indeed the dimer substates with wider angles are relatively rare (*SI Appendix*, Fig. S3 *C* and *D* and Scheme S2). Although it is not certain whether these very wide rotatory axis angle substates occur in native membranes, as the analysis was performed on isolated dimers, the data strongly suggest that the interfaces between monomers are significantly dynamic and that this dynamism is a general property of the interface, linked to motions of the enzyme that are both dependent and independent of catalysis. Indeed, this dynamism, which manifests as pseudoc2 symmetry about the dimer interface, necessitated specific refinement strategies to prevent the rotation of particle orientations about the pseudoc2-axis in order to isolate and subsequently maintain catalytically homogenous particle subsets during further particle realignment (*SI Appendix*, Scheme S1). Therefore, during image processing, each monomer was given the arbitrary designation “left or “right” with respect to a single viewing direction, and, for example, the state [s1:s2] and state [s2:s1] dimers are distinct and do not represent the same molecular state.

### Interactions between Dimers.

**Fig. 5.**
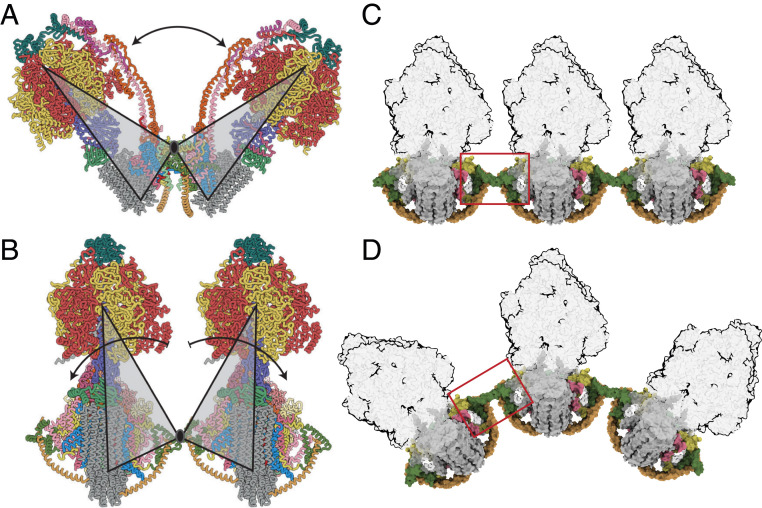
Accommodation of the dimeric ATP synthase to the membrane curvature in mitochondrial cristae along the axis of oligomerization via flexible interdimer contacts. (*A*) A view, orthogonal to the plane of the IMM, of the bovine ATP synthase dimer in state [s1:s1]. Gray triangles represent the monomers, moving back and forth as rigid bodies by rotation about their point of contact in j subunits; the rotatory axes of both monomers are aligned in the same plane, and the arrow indicates the range of angles between rotatory axes. (*B*) Side view, parallel to the plane of the IMM, of two monomers in a tetrameric arrangement of two adjacent dimers formed via contacts between g subunits. The arrows indicate the independent courses of their catalytic domains by rotation about this point. (*C* and *D*) Similar view to *B*, with the F_1_ domains in silhouette illustrating how changes in the interface between dimers (red box) allow a curved ultra-structure to develop along the apices of the cristae. For colors of subunits, see Fig. 1 legend.

**Fig. 6.**
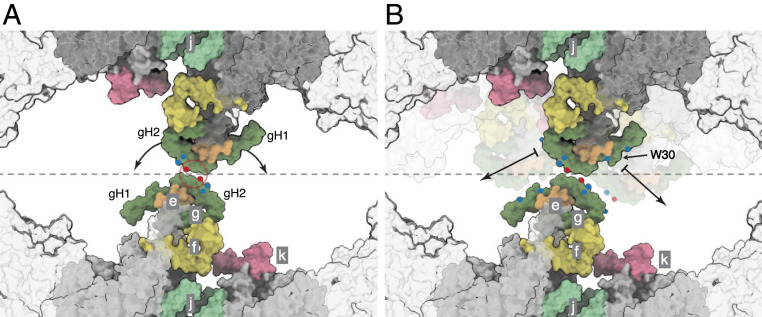
Close up of the dimer-dimer interface in a possible tetrameric bovine ATP synthase. The interactions shown as a molecular surface between g subunits in two adjacent dimers viewed from inside the mitochondrial matrix between the axes of the peripheral stalks, as in Fig. 7 *B*–*G*. Portions of the catalytic domains, situated above the viewing plane, are shown in gray transparency. The gray dashed line indicates the approximate tetramer interface, which is surrounded by lipids. (*A*) Rotation of the dimers relative to one another, as indicated by the arrows, about a contact point between the two g subunits, highlighted in the red circle. Key negative and positive polar residues are indicated by blue and red dots, respectively. (*B*) Contact dislocation of the interface between g subunits as indicated by the arrows. Possible positions of the translocated regions are shown in transparent underlay. Residue W30 might be involved in a hydrophobic “knobs-into-holes” interaction with regions of gH1 that are rich in leucine and isoleucine residues. Additional polar residues in gH1 and gH2 are indicated by colored dots. Subunits e, f, g, j, and k are khaki, straw yellow, forest green, sea-foam green, and pink, respectively. The c_8_ ring is gray.

**Fig. 7.**
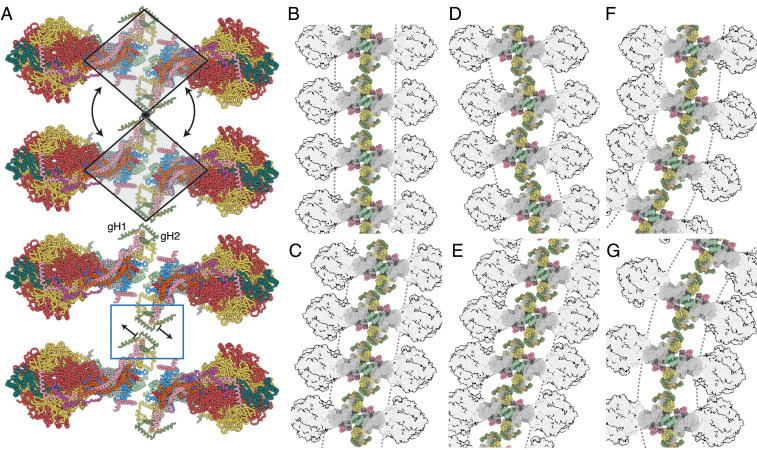
Possible modes of association of dimeric bovine ATP synthases in the cristae. (*A*) Row of dimers viewed from above the cristae tips. In the two upper complexes, the gray trapezoids represent individual dimers moving as rigid bodies; in the lower two complexes, the individual dimers are translated as indicated by the arrows via the dimer-dimer interface in the blue box. (*B–G*) The impact of translation or rotation of dimers on the long-range order of oligomeric rows. In *B*, back-to-face stacked rows of dimers provide the simplest architecture of straight and planar oligomers. (*C*–*E*) Effects of dislocation of the g-g interaction at the interface between two dimers. In *C* and *E*, compact back-to-face rows of dimers form with a range of inclinations with respect to the perpendicular axis, or, in *D*, as an alternating “zig-zag” arrangement. (*F* and *G*) Bending of the rows of oligomers laterally by rotation about a fixed point at the g-g contact. More complex arrangements can be envisaged by combination of the effects in *B–G*, with the additional possibility of curving the membrane positively along the axis of oligomerization, as in Fig. 5*D*. For colors of subunits, see Fig. 1 legend.

The issue of how dimeric ATP synthases interact with each other in the long rows that form along the apices of the cristae in the IMM cannot be resolved definitively by the current study of the structure of the isolated bovine dimers. However, the structures and other information suggest how the dimeric ATP synthases might reasonably form tetramers and higher oligomers involving homo-interactions between the two copies of each of subunits g and k. In the dimeric bovine structure, the 102-amino acid residue g subunit is folded into three α-helices, gH1 (residues 20–36), gH2 (residues 42–60), and gH3 (residues 69–93), with transmembranous gH3 augmenting the skeleton of wedge, and amphipathic gH1 and gH2 associated with the top of wedge on the matrix side of the IMM. In the dimeric complex, the two gH2s have the capability to contact each other across the dimer-dimer interface and link the dimers together, as depicted in Fig. 5, with weak interactions across the dimer-dimer interface between residues gQ50, gK53, and gK54 in each protomer (see Fig. 6), providing a potential molecular basis for the requisite sliding, translational, and rotational elements to generate the wide range of cristae structures that have been observed in mitochondria. Such a fluid interface would allow, for example, the long rows of ATP synthase oligomers to follow the curvature along the apices of the cristae (Fig. 5 *B*–*D*) and to readjust their positions as the curvature changes without impeding the dynamics of the cristae. This fluidity is illustrated in Fig. 5*B*, where the ATP synthase dimers follow the curvature of the IMM along the axis shown in Fig. 5 *C* and *D*. The fluidity of the dimer-dimer interface could allow either a rotation about a fixed contact point between g subunits or dislocate the contact and translate the dimers sideways (Fig. 7*A*). These adjustments of interdimer contacts, and combinations of them, can engender a range of oligomeric arrangements, which are inferred here from geometric considerations and the presentation of ATP synthase dimers in tomographic reconstructions of mitochondrial cristae. They include nonuniform packing (Fig. 7 *B*–*G*), where rotation about a fixed contact point produces lateral bends in oligomer rows (as viewed from above; see Fig. 7 *F* and *G*), and where contact dislocation permits the dimers to stack in a compact arrangement (Fig. 7 *C* and *E*) or form staggered rows (Fig. 7*D*). These arrangements are constrained geometrically in the cristae by the spherical catalytic domains of the enzyme. Thinning of the footprint of the membrane domain of the dimer, as viewed from above or below the membrane, resulting from changes in the angle between the rotatory axes as described in Movies S5 and S6, places adjacent catalytic domains in closer proximity and, therefore, limits the degree of lateral curvature of the oligomeric row. Such a mobile contact also accounts for the positive and negative curvature along the cristae rows (Fig. 5 *C* and *D*) and, in a variety of combinations with dislocations, produces zig-zagging, lateral bending, perpendicular curvature, and nonuniform packing. The oligomer rows may also deform plastically, much like an armature wire in a sculpture model, providing strength to maintain the apex of the cristae over long distances, simultaneously allowing different types of ultrastructure, such as lateral and perpendicular bends, to form. Many of these arrangements have been observed in tomographic reconstructions of the cristae (12, 17, 23).

It is also possible that subunit k could be involved in linking dimers together. During the process of assembly of the closely related human enzyme, this subunit is the last to be incorporated into complex, and in human cells grown under glycolytic conditions, subunit k turned over more rapidly than the other subunits of the enzyme, which turned over together at a slower rate (26). These observations led to the suggestion that subunit k might participate in a mechanism for remodelling IMMs (26). The partial structure of bovine k is compatible with this proposal. In the dimeric bovine complex, residues 12–47 of the 57-residue subunit k were resolved (2). The N-terminal region of the k subunit is folded into a short α-helix, kH1, from residues 14 to 17 and is linked to kH2 by a loop (residues 18–23). This region is followed by the single transmembrane α-helix, kH2, from residues 24 to 44 which is associated with subunit a via interactions with aH4 and aH5. The C-terminal region (residues 45–57) of subunit k is in the IMS and is predicted to be extended. Thus, it is possible that in the IMM, this extended region could be in contact with the C-terminal region of subunit g across the dimer-dimer interface. The distance between k and g subunits in adjacent dimers decreases as the angle between rotatory axes in the dimer increases. Rearrangement of wedge subunits along the dimer interface, concomitant with changes to the rotatory axes as shown in Movies S5 and S6, narrows the membrane footprint of the dimer, thereby bringing adjacent dimers closer.

### Influence of the Inhibitor Protein IF_1_.

Another mode of interaction of two ATP synthase dimers has been described where two inactive dimers are linked into tetramers of porcine (14) and ovine (27) ATP synthases by two dimeric IF_1_ molecules spanning between the globular F_1_ domains across the interface between the two dimeric ATP synthases. However, the membrane domains of the porcine tetramer were misinterpreted (2), but in a corrected structure (2), and in the structure of the ovine dimeric enzyme (27), the g subunits from adjacent dimers are in contact on top of the matrix side of the membrane via gH2 and the preceding loop (residues 39–53), the membrane domains of the e subunits are in close contact, and residues 13 and 14 of subunit k interact with gH1 on the matrix side of the membrane (2). In addition, its unresolved C terminus could interact with subunits g and a on the IMS side of the IMM. These tetrameric units require the catalytic states of the adjacent dimers to be [s1:s3] and [s3:s1] or vice versa.

The physiological relevance of this inactive tetramer is unclear. Both the porcine and ovine tetramers were purified from mitochondria isolated from animal hearts. Therefore, these experiments provide no information about whether the active dimers described here and these inactive tetramers coexist within the same mitochondrion. The coexistence of active dimers and inactive dimers within the same mitochondrion can be envisaged in two instances. First, individual cristae within a mitochondrion can have different membrane potentials (28). Therefore, both ATP synthesis by active dimers, and ATP hydrolysis followed by inhibition by IF_1_ and formation of tetramers, could happen at the same time in different cristae in the same mitochondrion. Second, if the prevailing physiological conditions led to activated IF_1_ dimers, but there was, despite its apparent abundance in mitochondria (29), insufficient active IF_1_ to inhibit the hydrolytic action of the dimeric ATP synthases, then this situation could lead to a mixture of active ATP synthase dimers in hydrolytic mode, and inhibited dimers within tetramers.

Independent of these physiological considerations, the structures of free dimers and equivalent dimers in tetramers differ substantially, and, for example, the angle between the rotatory axes of monomers in each dimer in tetramers is 112–116°, whereas it is ca. 76–95° in free dimers. This wider angle between monomers in dimers within tetramers is accompanied by an outward bending of each rotatory axis and a significant rearrangement of the interface between monomers in the dimers (the interface is compressed to allow close association of the catalytic domains, which is otherwise sterically prohibited by the g subunit). The membrane domain of the tetramer has undergone a structural rearrangement where the individual monomeric membrane domains appear to have collapsed inwards: See the change in the inclination of the c_8_ rings (*SI Appendix*, Fig. S4). Also, the tethering of adjacent catalytic domains by IF_1_ induces a torsion in the F_1_ and PS domains, tilting the rotatory axes accompanied by narrowing of the membrane domain in the dimeric unit (*SI Appendix*, Fig. S5). This change in the membrane domains could have arisen from the effects of extraction of the complexes from the membrane with detergents and the loss of endogenous lipids (*SI Appendix*, Fig. S4). Thus, it is possible that the binding of the two IF_1_ dimers to form the ATP synthase tetramers occurs during this extraction procedure, and that the formation of tetramers is an artifact. Relaxation of the tilted geometry of adjacent dimers and the dimer-dimer interface in the porcine and ovine tetramers is not possible because of the close proximity of the catalytic domains, and this prevents the formation of planar oligomeric rows. In contrast, the models of oligomerization of active dimers proposed here permit the formation of the variety of oligomeric arrangements needed to produce the geometry of the IMM. Also, they are compatible with the fluidity of the dimer interface observed in the structures of the bovine dimers (Fig. 4 and Movies S4–S7). These models of oligomerization of active dimers are incompatible with formation of inactive tetramers (*SI Appendix*, Fig. S6). The changes to the architecture of the membrane domains in the tetrameric structures produce a rigid curvature along the axis of oligomerization that is incompatible with the ultrastructures of the IMM observed by tomography (12, 17, 23), and the tetrameric structures inhibit the fluidity in oligomeric rows observed in vivo. Thus, this conformation is unlikely to be relevant in consideration of the wider plasticity of the IMM, especially as the in silico association of tetrameric complexes leads to a closed loop within six dimeric units (*SI Appendix*, Fig. S7).

One possible mode of binding of dimeric IF_1_ that has not been considered so far in the context of the possible influence of the inhibitor protein on the topography of the cristae is that one of its two active inhibitory regions could bind to an ATP synthase complex in hydrolytic mode, leaving the second inhibitory region unbound and unable to insert itself into a catalytic site in a second copy of the enzyme. Indeed, such a state might be quite common as the inhibitor could bind to all three of the possible states of the enzyme, s1, s2, and s3, and not be restricted to cross-linking adjacent s1 and s3 states. In contrast to the cross-linking of two dimers discussed above, such a binding mode would be unlikely to have a major impact on the ultrastructure of the cristae.

### Concluding Remarks.

Mammalian ATP synthases are highly conserved in the composition and sequences of their subunits, and, therefore, the nature of the association between monomers in the dimeric units will also be conserved. Likewise, the compositions and structures of the ATP synthases in yeasts and fungi resemble the mammalian enzymes in many respects (30, 31). In contrast, the structures of more distant ATP synthases, described recently (32–34), differ extensively from the mammalian enzymes. For example, their PS regions are much more complex and more rigid than those in mammals, and their cristae are likely to have very different properties to those described here. The elucidation of the molecular basis of the interactions of dimers of ATP synthases in the mitochondrial cristae of mammals, as well as those in more distant species, warrant further study by tomography of mitochondrial membranes.

## Materials and Methods

The purification of dimeric ATP synthase from bovine heart mitochondria, the preparation of cryo-em grids, data collection, preprocessing of micrographs, automated particle picking, and initial particle selection by two-dimensional classification has been described before (2). Particle data were sorted into subsets in which the catalytic state of each monomer in the pair was defined and classified into additional conformational substates within the subsets. Composite dimer models were constructed from monomeric models of bovine ATP synthase (2). For further details, see *SI Appendix*.

## Supplementary Material

Supplementary File

Supplementary File

Supplementary File

Supplementary File

Supplementary File

Supplementary File

Supplementary File

Supplementary File

## Data Availability

Protein models and electron density data have been deposited in the Protein Data Bank and the Electron Microscopy Data Bank under the following accession nos.: 7AJB (EMD-11428), 7AJC (EMD-11429), 7AJD (EMD-11430), 7AJE (EMD-11431), 7AJF (EMD-11432), 7AJG (EMD-11433), 7AJH (EMD-11434), 7AJI (EMD-11435), 7AJJ (EMD-11436), EMD-11449–EMD-11454, EMD-11460–EMD-11466, EMD-11472–EMD-11477, EMD-11479, EMD-11480, EMD-11484–EMD-11487, EMD-11499–EMD-11512, EMD-11527–EMD-11546.
