# Supplementary material for: Interface mobility between monomers in dimeric bovine ATP synthase participates in the ultrastructure of inner mitochondrial membranes
Source: Proc Natl Acad Sci U S A. 2021 Feb 4;118(8):e2021012118. doi: 10.1073/pnas.2021012118 (PMC7923604; doi:10.1073/pnas.2021012118)
Supplement: Supplementary File [file pnas.2021012118.sapp.pdf]

PNAS
www.pnas.org

Supplementary Information for

Interface mobility between monomers in dimeric bovine ATP synthase participates in the ultrastructure of inner mitochondrial membranes

Tobias E. Spikes, Martin G. Montgomery and John E. Walker
The Medical Research Council Mitochondrial Biology Unit, University of Cambridge, Cambridge Biomedical Campus, Hills Road, Cambridge CB2 0XY, United Kingdom

Corresponding author: John E. Walker
Email: walker@mrc-mbu.cam.ac.uk

This PDF file includes:

Supplementary text
Figures S1 to S7
Tables S1 to S3
Legends for Movies S1 to S7
SI References

Other supplementary materials for this manuscript include the following:

Movies S1 to S7

Supplementary Materials and Methods

Purification of dimeric bovine ATP synthase. The dimers were purified in the presence of glycodiosgenin, Brij-35 and phospholipids, as described elsewhere (1).

Cryo-EM data collection and processing. Grids for cryo-em containing the dimeric bovine ATP synthase were prepared and imaged by high-resolution cryo-electron microscopy as described before (1). The particle data were sorted into sub-sets in which the catalytic state of each monomer in the pair was defined (Scheme S1) and then further classified to reveal additional conformational sub-states within them (Scheme S2 and Fig. S2). The pre-processing of micrographs, automated particle picking and initial particle selection by 2D classification have been described elsewhere (1) and are summarized below in Scheme S1. Image processing procedures was performed with RELION-3.0 (2–6).

Fitting of models to density. Composite dimer models were constructed from published monomeric models of bovine ATP synthase (1), which were built and refined into focussed locally refined cryo-em reconstructions of higher resolution using COOT (7) and Phenix (8, 9) (see Table S1 and S2). They were rigid body fitted into reconstructions of the dimeric enzyme produced according to Schemes S1 and S2 using UCSF ChimeraX (10) and COOT (7). The surfaces of cryo-em reconstructions were colored according to the subunit assignments in composite dimer models as in Movies S3, S4, S5 and S6. An octomeric assembly of porcine ATP synthase (Fig. S4) was made from a re-interpreted model of porcine ATP synthase (PDB6ZNA (1), EMBD-0667 (11)).

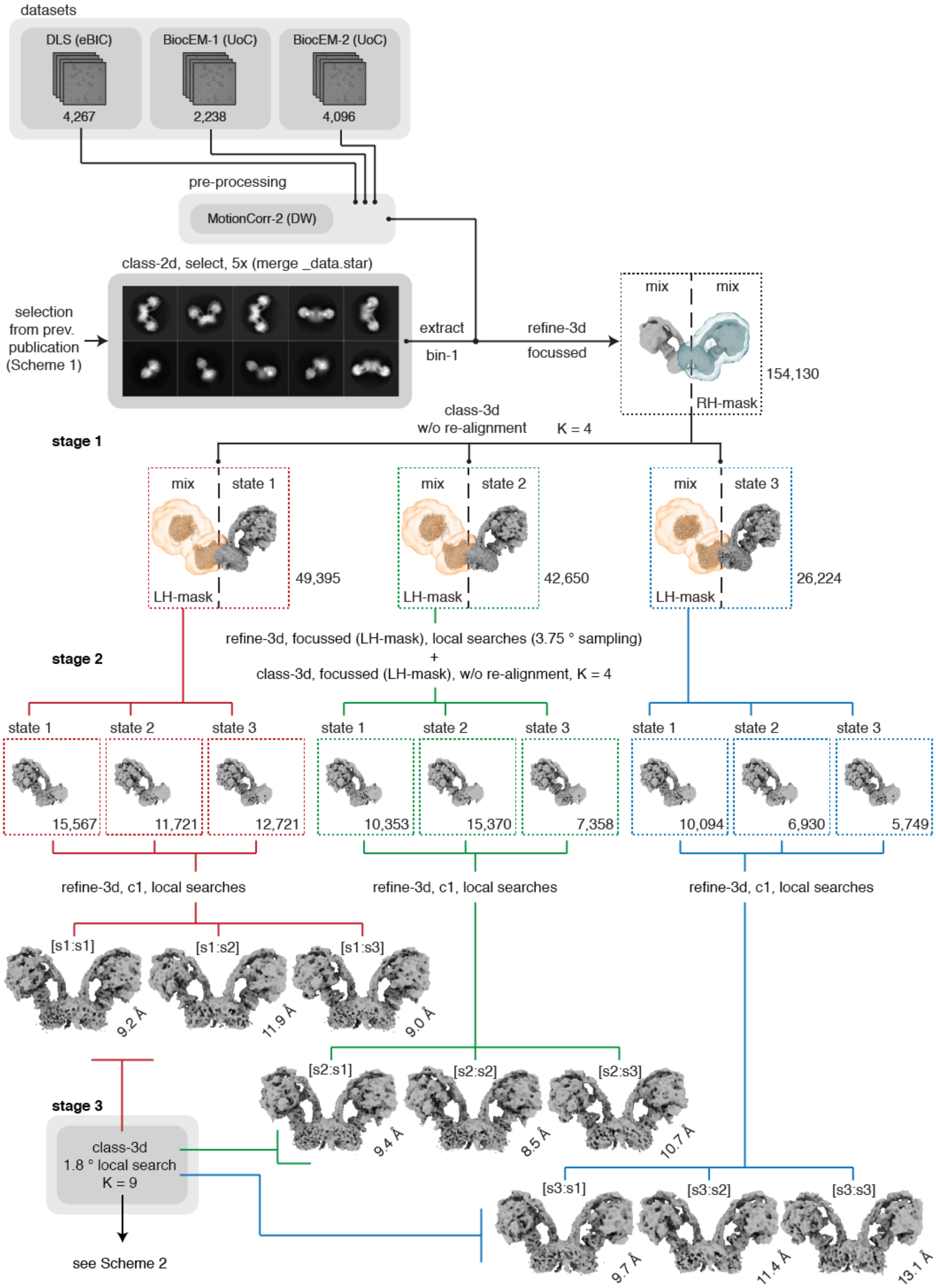

Scheme S1. Enumeration of all catalytic state combinations in dimeric bovine ATP synthase. Because the dimer particles in the data-sets were heterogeneous, high resolution structures of the intact dimer could not be determined by refinement of the whole particle set with or without the imposition of c2 symmetry. Therefore, consensus reconstructions represent an average of two distinct types of heterogeneity arising, first from the positions of the asymmetrical F_1-domain

catalytic relative to the PS in the monomeric complex, which are independent of one another, and second from variance in the spatial relationship between monomers arising from differences in the angle between the rotatory axes of the two F_1-c_8 domains. Therefore, a hierarchical classification strategy employing three stages was applied. First, the catalytic states of each half of the assembly were resolved independently, in order to provide consensus dimer reconstructions in each of the nine combinations of rotational state (shown above), Then they were resolved into classes representing distinct structural conformations with defined catalytic states (Scheme S2). Raw movie frames were corrected for motion, and were dose-weighted to improve the reconstructions in lieu of particle polishing of many individual sub-sets. 154,130 dimer particles, selected by 2D classification in a previous work (1), were re-extracted from the dose-weighted micrographs at a sampling rate of 1.048 Å/pix and separated into rotational states of the right monomer by refining the particles whilst employing a monomeric mask (blue) against the consensus dimer reference, and subsequently classifying without particle re-alignment (stage 1). This procedure yielded 49,395 particles in state-1, 42,650 particles in state-2, 26,224 particles in state 3 and 39,891 particles in a fourth class that were discarded. Each of the three classes representing the rotational states of the right molecule were refined against the consensus dimer reference with a mask encompassing the left monomer (orange). Rotation of the particles about the pseudo-twofold rotational symmetry axis was prevented by local angular searches from the orientations determined for the right monomer, thereby maintaining the relative positions of the right and left monomers with respect to the refinement reference. The left monomer was classified into the three rotational states without particle re-alignment (stage 2) from the refined orientations, leading to particle sub-sets where the rotational state of each monomer in the pair is defined, albeit with a heterogeneous relationship between their spatial arrangement in the overall assembly. The colored dashed boxes contain the number of particles in each sub-state. During this process, an additional 22,826 particles in the fourth class failed to separate into a distinct rotational state and were discarded. As before, rotation of the particles about the pseudo-c2 symmetry axis was prevented by refinement of particle sub-sets without symmetry and local searches from the previously determined orientations. These structures are shown at the bottom of the scheme and are named according to the catalytic state of the right monomer, followed by the left monomer (i.e. the order in which they were classified) as follows; states [s1:s1], [s1:s2], [s1:s3], [s2:s1], [s2:s2], [s2:s3], [s3:s1], [s3:s2] and [s3:s3] at resolutions of 9.2, 11.9, 9.0, 9.4, 8.5, 10.7, 9.7, 11.4, and 13.1 Å, respectively, with applied B-factors ranging from -215 to -500 $Å^2$. In stage 3, each particle set was classified further with reference to its respective consensus structure without particle re-alignment into nine classes (see Scheme S2).

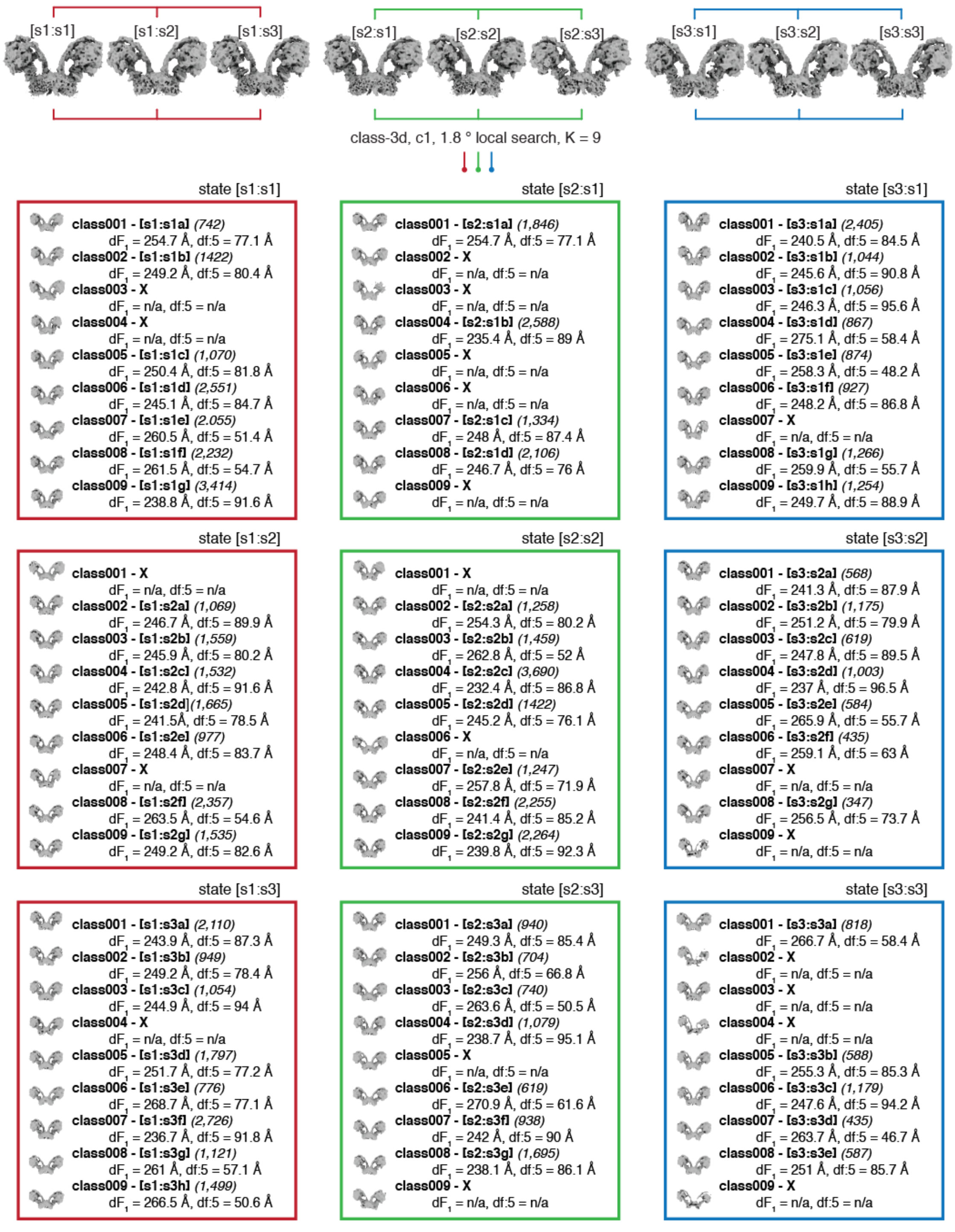

Scheme S2. Identification and analysis of structural heterogeneity in dimeric bovine ATP synthase. Each particle set represents the dimers in defined catalytic states (see Scheme S1). Local searches from the consensus orientations for alignment were enforced and the dimer particles in each set were classified into nine additional classes, shown in the colored boxes, right monomers in state 1, state 2 and state 3 in red, green and blue, respectively. The composite atomic models for the corresponding rotational state in each half of the molecule were rigid body fitted into the reconstructions. Two centroids, representing the catalytic domain and residue 5 of subunit f in a small matrix protrusion, were calculated in each monomer and the distances between them in each dimer were measured. This pair of distances, dF_1 and df:5, serve as proxy for measurements

of the angle between the rotatory axes. These measurements in Ångströms accompany the number of particles (brackets) and a map identifier (square brackets). The per-particle distributions of the measurements are summarized in Fig. S3. Twenty-two maps, with too few particles or with obvious artefacts and denoted with an "X", were excluded from this analysis, leaving fifty-nine reconstructions (see Table S2).

Analysis of sequences of subunits. Pairwise comparisons of the sequences of orthologous subunits of bovine, porcine and yeast ATP synthases subunits were made with CLUSTAL O (12) on www.uniprot.org, and their secondary structures were predicted with PSIPRED (13, 14).

Production of Figures, movies and animations. Figures and movies were prepared with USCF ChimeraX (10).

Movie S1. Structural heterogeneity in dimeric bovine ATP synthase associated with catalysis. Two copies of the atomic model of the bovine membrane domain (6ZBB) were rigid body fitted into the cryo-em density of the state [s2:s2] consensus dimer reconstruction and a simulated 7 Å density was produced from both chains of subunits a and j. Then, each of the other consensus dimer reconstructions produced by stage 2 of the hierarchical classification procedure (Scheme S1) were fitted to this simulated density so that the maps were aligned by the membrane domains, and the movie was produced by cross-fading between the different reconstructions in the following order; state [s1:s3], [s3:s1], [s1:s2], [s2:s3], [s3:s2], [s2:s1], [s1:s1], [s2:s2], [s3:s3], [s1:s1].

Movie S2. The rotary cycle during synthesis and hydrolysis. This movie was produced in the same manner as Movie S1 except that the composite atomic model of the corresponding rotational state, PDB6ZPO, PDB6ZQM and PDB6ZQN (1) for states 1, 2 and 3, respectively, in each monomer was fitted into the consensus reconstruction and the surface of each subunit was colored accordingly.

Movie S3. Pivoting of the membrane domains of adjacent monomers of bovine ATP synthase about the matrix contact between j-subunits during catalysis. This movie was produced in the same manner as Movies S1 and S2 except that the composite atomic model of the corresponding rotational state, PDB6ZPO, PDB6ZQM and PDB6ZQN (1) for states 1, 2 and 3, respectively, in each monomer was fitted into the appropriate consensus reconstruction and the surfaces of the cryo-em densities were hidden. Side chains were hidden from the atomic model, and the secondary structure elements were dilated in appearance. The movie was produced by cross-fading between the different models in the order state [s1:s2] > [s2:s3] > [s3:s1] > [s1:s2] and as described in the legend of Movie S3.

Movie S4. The fluidity, independent of catalysis, of the monomer:monomer interface at rotatory axis angles less than or equal to 90°. Composite atomic models of the bovine ATP synthase monomer in rotational state 2 (PDB6ZQM (1)) were rigid body fitted into the dimer sub-state [s2:s2c] reconstruction, in which the angle between rotatory axes is *ca.* 76°, produced by stage 3 of the hierarchical classification (see Schemes S1 and S2). A simulated 7 Å density was created from the model of the left monomer (with respect to the screen viewing direction) to which were fitted the reconstructions of the [s2:s2f] and [s2:s2g] dimer sub-states, aligning the reconstructions to a single monomer. Copies of the state 2 atomic model (PDB6ZQM (1)) were fitted into the remaining monomer domains and the surfaces of individual subunits were colored according to their position in the atomic model.

Movie S5. Trajectory, independent of catalysis, towards the formation of a wide angle between the central axes of the F_1-c_8 domains in a dimer of ATP synthase. Composite atomic models of the bovine ATP synthase monomer in rotational state 2 (PDB6ZQM (1)) were rigid body fitted into the sub-state [s2:s2c] reconstruction, in which the angle between rotatory axes is *ca.* 76°,

produced by stage 3 of the hierarchical classification (see Schemes S1 and S2). A simulated 7 Å density was created from both chains of subunit a and subunit j to which were fitted the reconstructions of the [s2:s2a], [s2:s2e] and [s2:s2b] dimer sub-states, to align them by their membrane domains. Then each reconstruction was re-sampled onto the coordinate system of the [s2:s2c] sub-state and the densities were interpolated in the order sub-state [s2:s2c] > sub-state [s2:s2a] > sub-state [s2:s2e] > sub-state [s2:s2b]. Copies of the state 2 composite atomic model (PDB6ZQM (1)) were fitted into the new positions of the sub-state reconstructions, and the positions of the atomic coordinates of each model were similarly interpolated over an equal number of frames, in the same order. In each frame of the interpolated trajectory, the surfaces of subunits were colored in the reconstructions.

Movie S6. Detailed view of the interface rearrangement, independent of catalysis, arising in the trajectory towards the wide angle dimeric ATP synthase. Top view, as viewed from the matrix, of subunit a and residues 19-49 of subunit j progressing along the interpolated atomic model trajectory from sub-state [s2:s2c] > [s2:s2a] > [s2:s2e] > [s2:s2b]. The movie was prepared as described in the Supplementary Information, Production of Movies, for Movie S5.

Movie S7. Catalytic and structural heterogeneity in purified dimeric bovine ATP synthase. All sub-states identified by the hierarchical classification strategy described in Scheme S2 are shown. They were fitted manually to one another by aligning them approximately via their membrane domains. They are cycled in alphanumerical order starting from sub-state [s1:s1a].

Supplementary Figures

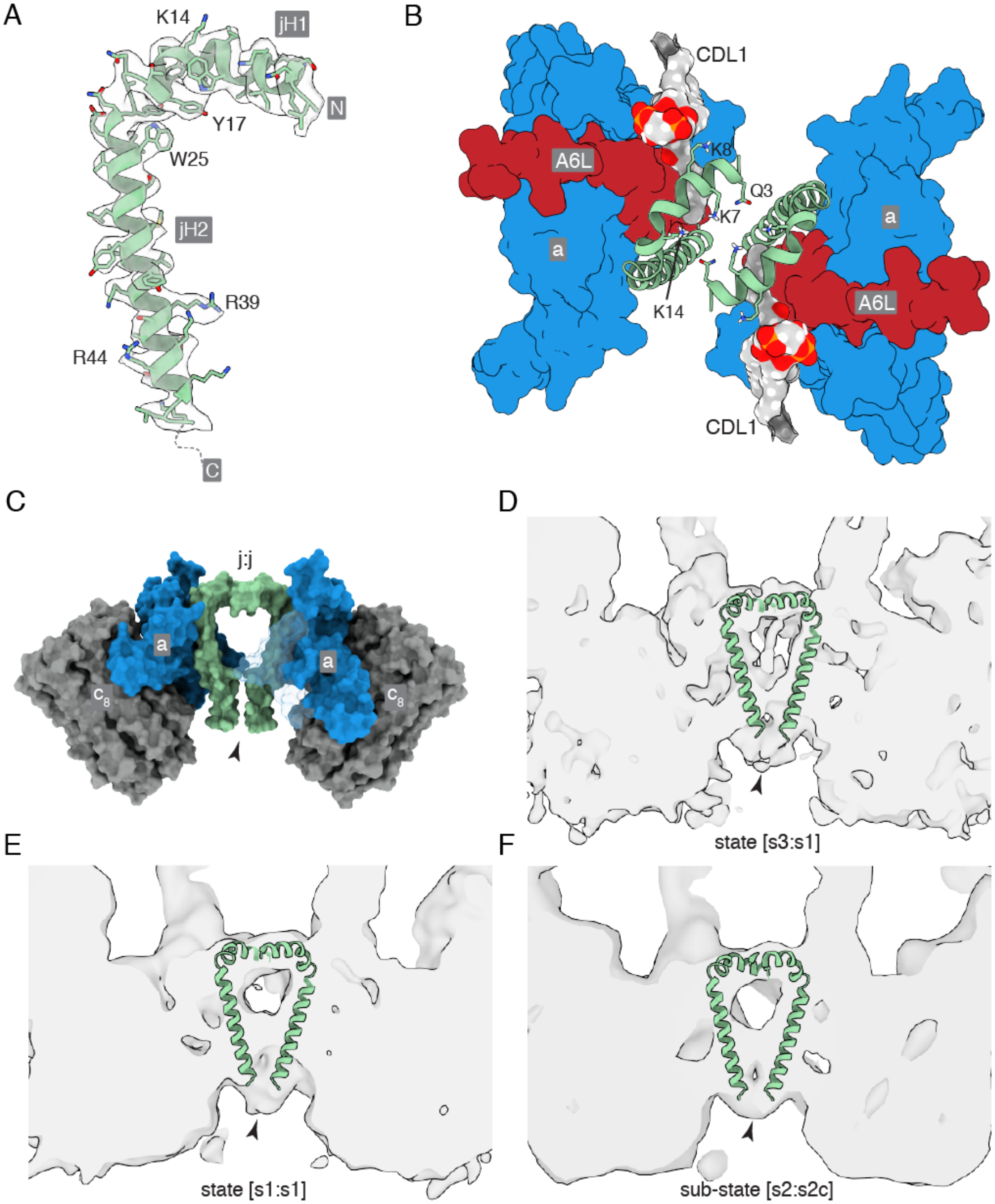

Fig. S1. The topology of subunit j in dimeric bovine ATP synthase. *A,* side view of the cryo-em density of the subunit j (grey transparency, EMD-11149) and the fitted atomic model (sea-foam green, PDB6ZBB)(1). Residues used to determine the sequence register during model building and their corresponding densities are labelled. The N-terminus of subunit j is folded into an amphipathic α-helix jH1 (residues 1-20), which lies in the lipid head group region on the matrix side of the IMM, followed by the transmembranous jH2 which lies adjacent to A6L and is associated with subunit a. The C-terminal region of jH2 (residues 40-49), protrudes into the IMS, indicated in *C* by the arrowhead; *B*, top view, from inside the mitochondrial matrix between the two peripheral stalks of the dimer (not shown), with the a-subunit (cornflower blue) and A6L-subunit (brick red) shown as a

molecular surface and the j-subunit (sea-foam green) in cartoon. The negatively charged headgroup of CDL1 (grey surface, colored by heteroatom) is bound to jK8, and to residues fQ38 and fY42 of the f-subunit in the wedge (not shown) and residues aT33, and A6LK27 and A6LK30. Residues jQ3, jK7 and jK14 are shown projecting toward the interface; *C*, side view, rotated 90° into the plane of the paper with respect to *B*, of the molecular surfaces of the a-subunit (cornflower blue) and the j-subunit (sea-foam green), and the c_8-ring (grey), in the dimeric membrane domain of the bovine ATP synthase; *D-F,* cross-sectional side views of three bovine ATP synthase dimers that exemplify density, attributed to the C-terminal regions of the j subunits, beyond the modelled residues. The state [s3:s1], state [s1:s1] and sub-state [s2:s2c] dimers are shown in *D*, *E* and *F*, respectively. The modelled regions of the j-subunits are overlayed onto the transparent density.

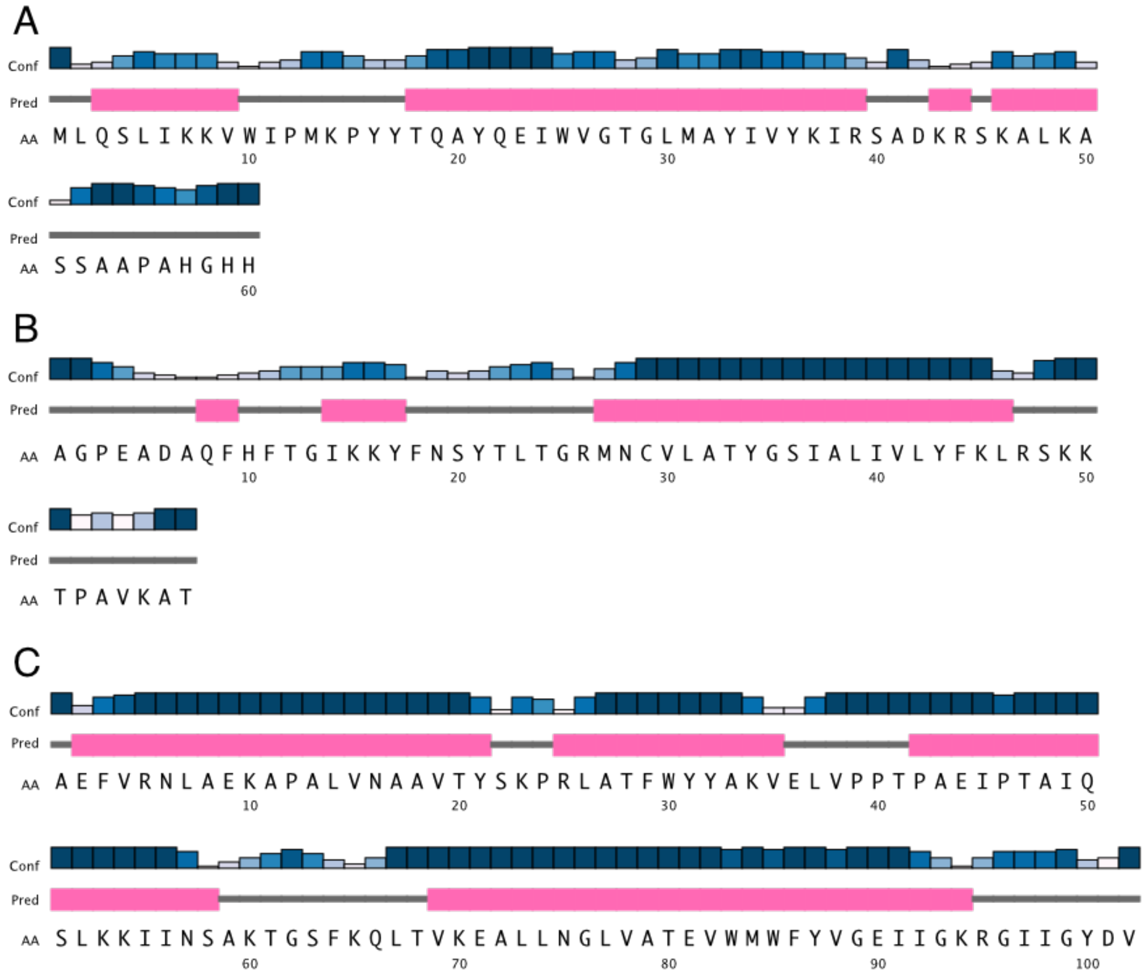

Fig. S2. The predicted secondary structures of subunits j, k and g of bovine ATP synthase. In *A*, *B* and *C*, the sequences of subunits j, k and g, respectively, are given in single letter amino acid code, and their predicted secondary structure elements and per-residue confidence scores are shown, respectively, in the lower, middle and upper rows of each panel. α-Helices are pink, and extended structures grey. Confidence scores are shown on a white-blue color scale, with dark blue being the most confident. The secondary structure predictions were performed with PSIPRED (14, 15). In the structure of bovine subunit j, residues 1-20 form an amphipathic α-helix (jH1) that lies in the plane of the IMM side leaflet and residues j22-39 form the transmembrane region of jH2. The C-terminal region of jH2 (residues 40-49) protrudes into the IMS. The rest of the sequence, comprising residues j50-60, was not modelled but is predicted to form an extended structure as shown above.

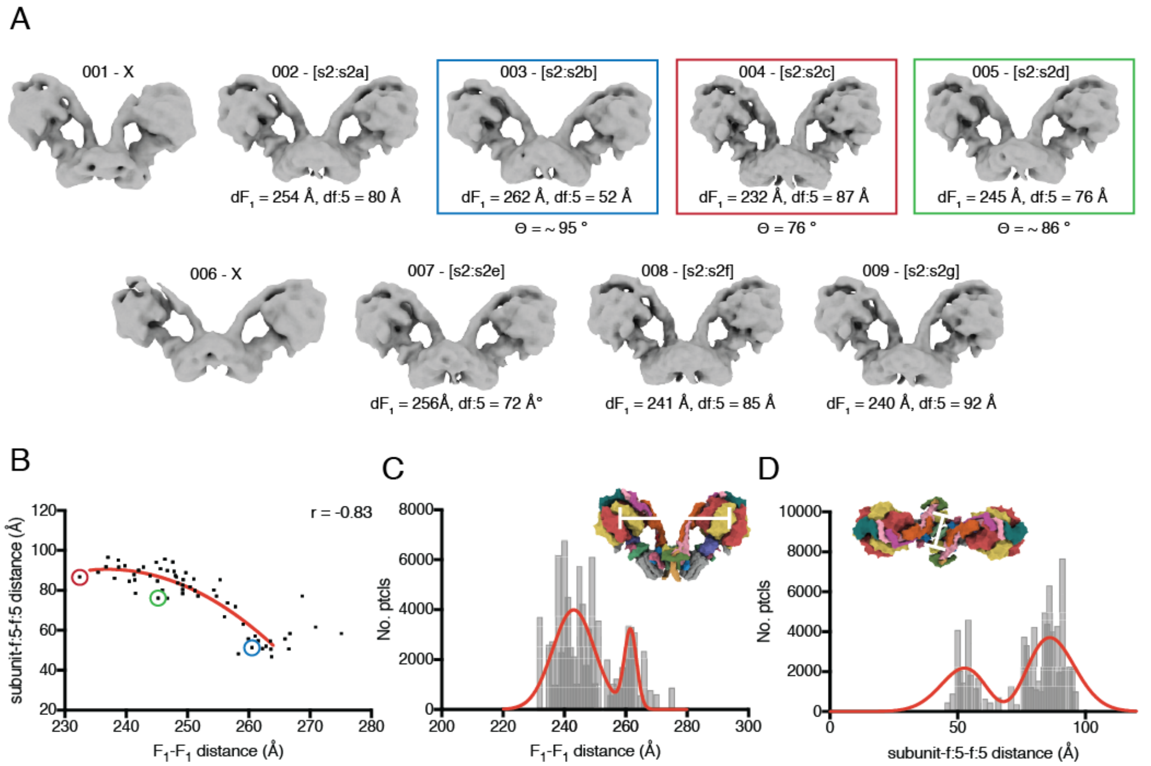

Fig. S3. Quantitation of the structural heterogeneity observed in dimeric bovine ATP synthase. *A*, stage 3 of the hierarchical classification (see Schemes 1 and 2) for dimer particles in catalytic state [s2:s2]. Particles in class 001 and 006 were discarded; Movies S3, and S4 and S5, are derived from reconstructions of sub-states [s2:s2c], [s2:s2f] and [s2:s2g], and [s2:s2c], [s2:s2a], [s2:s2e] and [s2:s2b], respectively; *B*, correlation between the distance between F_1-domains and the width of the membrane. Encircled dots correspond to the maps in colored boxes in *A*. As the angle between the rotatory axes increases, the catalytic domains separate requiring the dimer interface to re-arrange, leading to the membrane domain becoming narrower in the direction perpendicular to the plane of the rotatory axes (as in *D, Inset*). *C* and *D*, distribution of the distances between F_1-domains and between residues 5 in f-subunits, respectively. The most abundant inter-F_1 distances clustered at *ca.* 240 Å, and the distance between residues 5 in f-subunits was *ca.* 80-90 Å, which corresponds to a range of angles between rotatory axes of *ca.* 76-86°. In *C* and *D*, the insets show the approximate points of measurement.

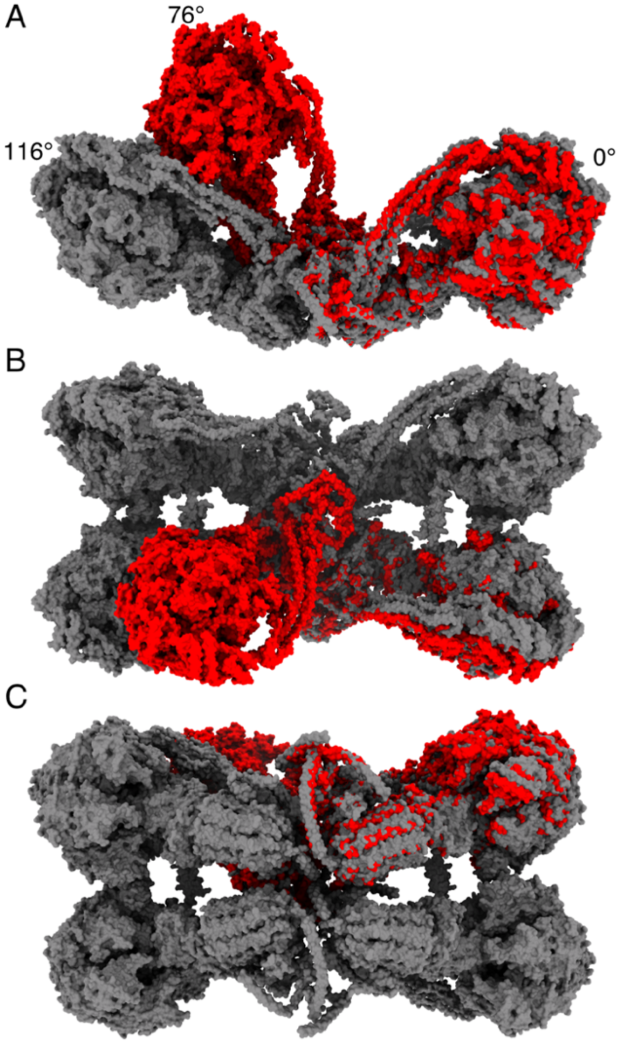

Fig. S4. Comparison of the angles between monomers in bovine dimers and porcine tetramers of ATP synthase. The porcine tetramer (grey, PDB6J5K) consists of the state [s1:s3] and [s3:s1] dimers. The model of the bovine state [s1:s3] dimer (red, PDB 7AJD) has been superimposed onto one of the s3 monomers of the porcine tetramer via their a-subunits. The models are displayed in surface representation. *A, B,* and *C,* views along the plane of the membrane, from above, and below, respectively. The membrane subunits of the porcine enzyme appear to be folding in on themselves probably because the removal of lipids during extraction of the complex with detergent causes a rearrangement of the membrane subunits thereby increasing the angle between monomers. A similar comparison cannot be made with the ovine tetramer as neither the map nor the model is publicly available.

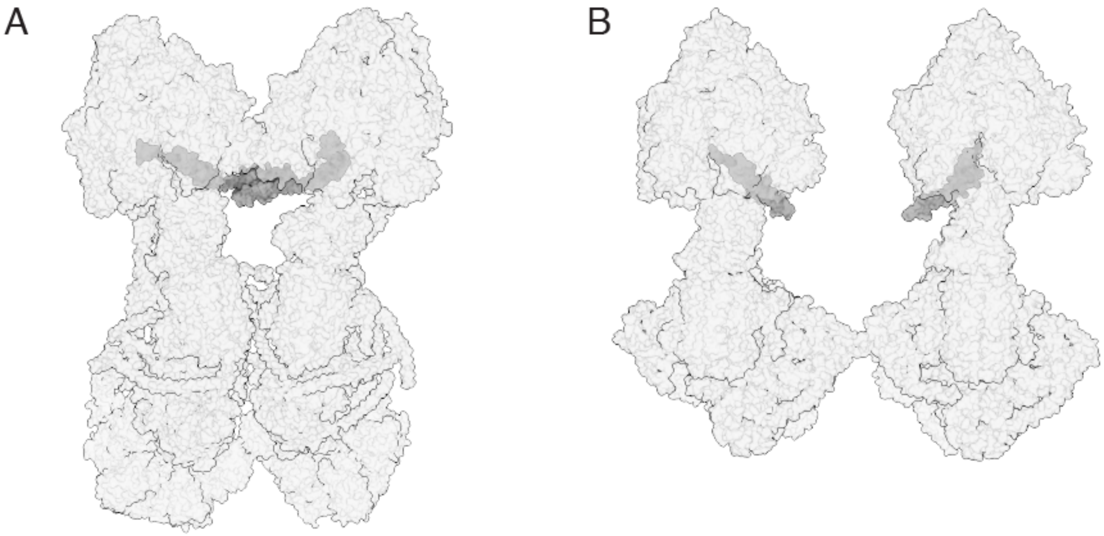

Fig. S5. Comparison of the arrangement of adjacent dimers in tetramers of porcine and bovine ATP synthase. *A,* side view of the deposited porcine tetramer model (PDB6J5K) (11) in which a pair of monomers in two adjacent dimers are oriented in the viewing plane. Inhibitor protein IF_1 is shown in solid grey and all other subunits are in grey transparency. The second pair of monomers projects away from the viewing plane. In this tetrameric arrangement, each adjacent catalytic domain is linked by a dimer of IF_1. This tethering of catalytic domains reduces the distance between the monomers and gives rise a significant tilt in the rotatory axes. It appears from the deposited structure that this tilting requires a significant rearrangement of the membrane domain in the region of the dimer-dimer interface. *B,* an equivalent view of a hypothetical bovine tetramer in the planar oligomeric arrangement suggested in Fig. 6B. This figure was generated by approximately placing models of the state [s1:s3] and [s3:s1] dimers (PDB7AJD and PDB7JH, respectively) according to the dimer-dimer interface suggested in Fig. 6A, with a small contact between adjacent regions of gH2. This distance represents the furthest point between dimers that could maintain a labile association in an oligomeric row. Note that this arrangement increases the distance significantly between each of the β_{DP}-subunits which are part of the $\alpha_{DP}\beta_{DP}$-catalytic interface that binds I1-60. See also *SI Appendix* Fig. S7.

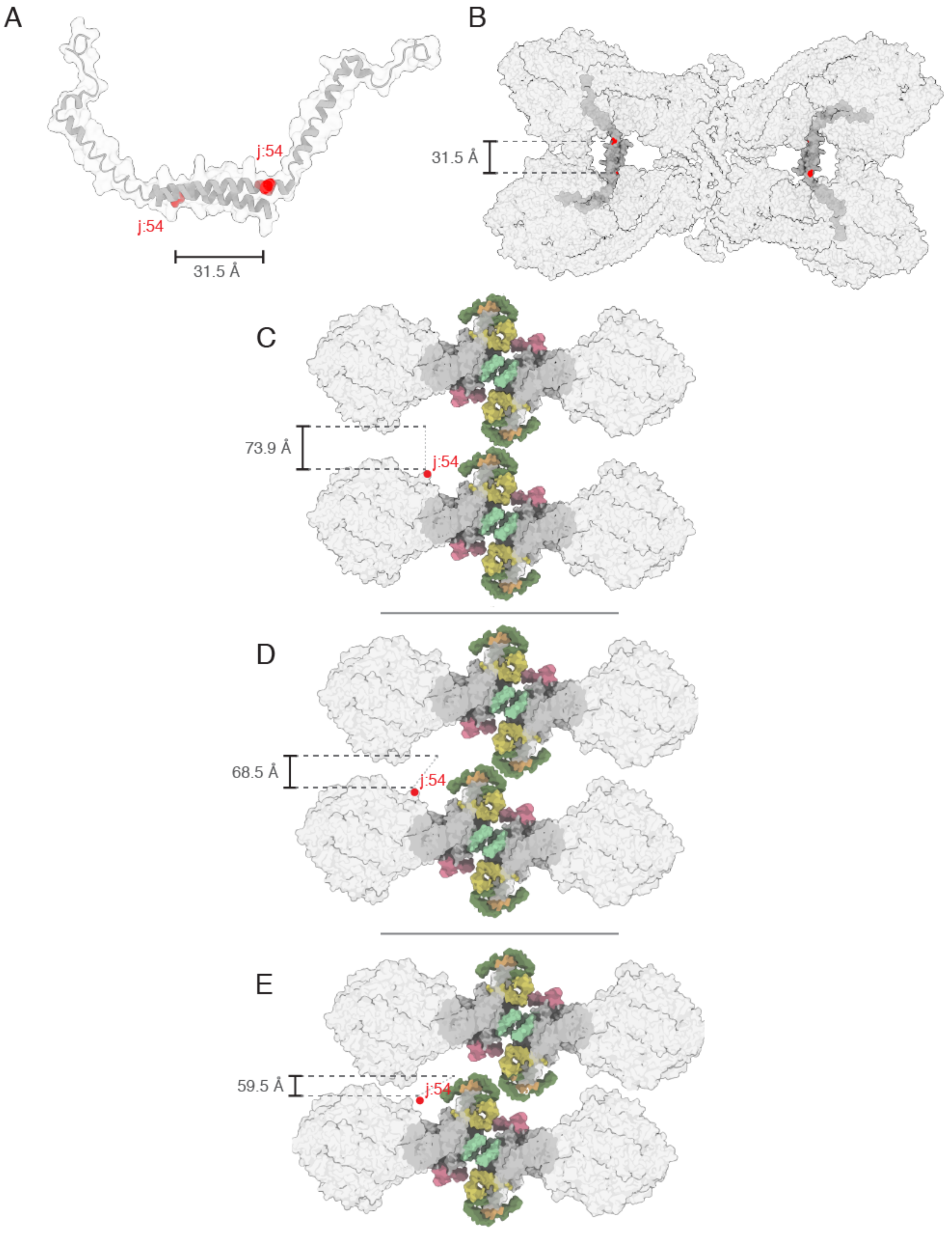

Fig. S6. Comparison of the inter-dimer distances between residues 54 of IF$_1$ in tetramers of porcine and bovine ATP synthase. *A,* the atomic model of dimeric porcine IF$_1$ (PDB6J5K) (11) shown in grey cartoon with a transparent overlay of the solvent excluded surface. Residues 54 are colored red and the distance between them is given in Ångstroms. *B,* a view from within the matrix and between the peripheral stalks of the atomic model of the porcine tetramer (PDB6J5K) showing

the *in situ* arrangement of the IF_1 dimer. IF_1 is shown in solid grey and all other subunits are shown in grey transparency. *C,* the same view of the proposed bovine tetramer in the planar oligomeric arrangement suggested by Fig. 6B. Models of the state [s1:s3] and [s3:s1] dimers (PDB7AJD and PDB7JH, respectively) were placed approximately according to the 2D arrangement in Fig. 6B, and the distance between residue 54 in each I1-60 molecule was measured in ChimeraX. The distance is indicated in Ångstroms. *D,* the tetrameric arrangement and I1-60 distance according to the oligomeric arrangement in Fig. 6C or D. *E,* the tetrameric arrangement and I1-60 distance according to the oligomeric arrangement in Fig. 6E. Note that reducing the distance between adjacent catalytic domains in this manner, that is, without rearrangement of the dimeric membrane domain, results in a translation of one dimer with respect to the other and similarly translating the position of the protruding inhibitor protein (light grey dotted line). Thus, these oligomeric arrangements are incompatible with the binding of dimeric IF_1 in absence of a significant rearrangement of the membrane domain or of the ultrastructure of the inner membrane. As detailed in Fig. S3, those dimeric structures with wider angles between their rotatory axes have correspondingly narrower membrane domain footprints, and this effect could permit closer stacking of oligomers. However, as noted in the Discussion section, it is highly unlikely that inactive tetramers and active dimers (and oligomers and so on) co-exist within a single mitochondrial crista.

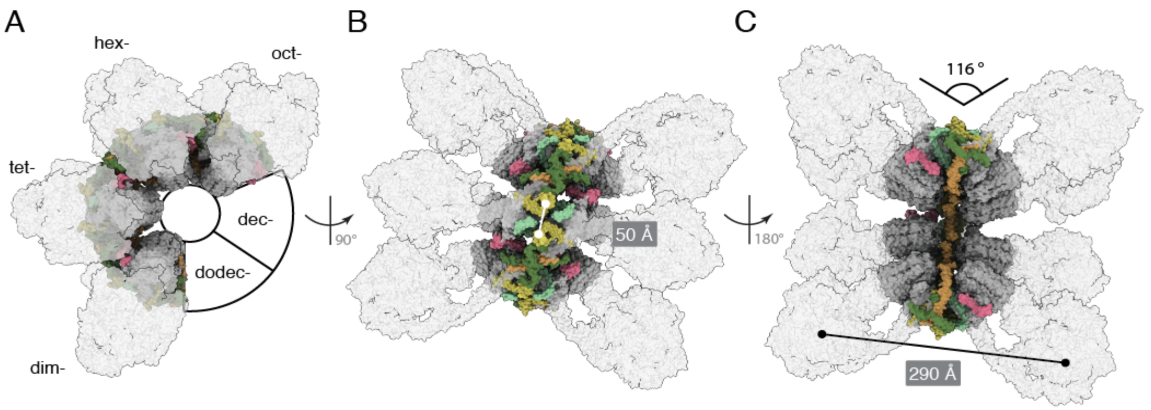

Fig. S7. Model of oligomeric porcine ATP synthase. An octomer of the reinterpreted porcine ATP synthase (PDB6ZNA) (1) was constructed by aligning two overlapping tetramers with one another and repeating the process for four dimeric units whilst maintaining the spatial relationship observed in the reported tetrameric assembly reconstruction (EMB-0667) (11). *A*, side view of the oligomer which forms a closed loop within six dimeric units if the compact tetramer interface is maintained strictly. We suggest that the formation of a tetramer and therefore of higher oligomers, in the presence of dimeric IF_1 and the absence of a native membrane environment, in this way also contributes to the large F_1-F_1 distance, narrow of the membrane domain and very wide rotatory axis observed in the structure of the porcine tetramer. These factors are closely related to the specific membrane domain organization at the interface between monomers. *B*, top view of the oligomer, with the subunit-f:5-subunit-f:5 distance labelled. *C*, a 180° rotated view of *C* with the F_1-F_1 distance labelled. These measurements can be compared with those in Scheme 2 and Fig. S2 as a proxy for the rotatory axis angle, which was measured as *ca.* 116° by the method described in Fig. 1, but was not measured for all bovine sub-states.

Table S1. Published structures employed in this work

	PBD	EMDB	Description	Reference
**State 1**	6YY0	EMD-11001	Bovine ATP synthase catalytic and rotor domains in rotational state 1	a
**State 2**	6Z1R	EMD-11039	Bovine ATP synthase catalytic and rotor domains in rotational state 2	a
**State 3**	6Z1U	EMD-11040	Bovine ATP synthase catalytic and rotor domains in rotational state 3	a
**F_o**	6ZBB	EMD-11149	Bovine ATP synthase monomeric membrane domain	a
**State 1 composite**	6ZPO	EMD-11342	Complete bovine ATP synthase monomer in rotational state 1	a
**State 2 composite**	6ZQM	EMD-11368	Complete bovine ATP synthase monomer in rotational state 2	a
**State 3 composite**	6ZQN	EMD-11369	Complete bovine ATP synthase monomer in rotational state 3	a
**Porcine F_o**	6ZMR	EMD-0668	Porcine ATP synthase monomeric membrane domain, reinterpreted	a, b
**Porcine F_o tetramer**	6ZNA	EMD-0667	Porcine ATP synthase tetrameric membrane domain, reinterpreted	a, b

a, Spikes *et al*, 2020; b, Gu *et al*, 2020

Table S2. Deposited data-sets relating to the structure of the dimeric bovine ATP synthase

	EMDB	PDB	Resolution (Å)	B-factor ($Å^2$)	Num. particles	Detail	Comment
**State [s1:s1]**	EMD-11428	7AJB	9.20	-300 [1]	15,567	Hierarchical classification of all dimer particles (Scheme 1)	Consensus, catalytically homogenous, structurally heterogenous
**State [s1:s2]**	EMD-11429	7AJC	11.9	-300 [1]	11,721	〃	〃
**State [s1:s3]**	EMD-11430	7AJD	9.00	-260 [1]	12,721	〃	〃
**State [s2:s1]**	EMD-11431	7AJE	9.40	-255 [1]	10,353	〃	〃
**State [s2:s2]**	EMD-11432	7AJF	8.45	-215 [1]	15,370	〃	〃
**State [s2:s3]**	EMD-11433	7AJG	10.7	-224 [1]	7,358	〃	〃
**State [s3:s1]**	EMD-11434	7AJH	9.70	-350 [1]	10,094	〃	〃
**State [s3:s2]**	EMD-11435	7AJI	11.4	-265 [1]	6,930	〃	〃
**State [s3:s3]**	EMD-11436	7AJJ	13.1	-500 [1]	5,749	〃	〃
**Sub-state [s1:s1a]**	EMD-11448		20.1	unsharpened [2]	742	Classification of state [s1:s1] consensus particles, K = 9, class001 (Scheme 2)	wide rotatory axis angle, subunit j not resolved
**Sub-state [s1:s1b]**	EMD-11449		17.5	〃	1,422	Classification of state [s1:s1] consensus particles, K = 9, class002 (Scheme 2)	intermediate rotatory axis angle, subunit j partially resolved
**Sub-state [s1:s1c]**	EMD-11450		18.1	〃	1,070	Classification of state [s1:s1] consensus particles, K = 9, class005 (Scheme 2)	narrow rotatory axis angle, subunit j C-terminus resolved

**Sub-state [s1:s1d]**	EMD-11451	14.9	〃	2,551	Classification of state [s1:s1] consensus particles, K = 9, class006 (Scheme 2)	intermediate rotatory axis angle, subunit j C-terminus resolved
**Sub-state [s1:s1e]**	EMD-11452	16.4	〃	2,055	Classification of state [s1:s1] consensus particles, K = 9, class007 (Scheme 2)	wide rotatory axis angle, subunit j not resolved
**Sub-state [s1:s1f]**	EMD-11453	15.9	〃	2,232	Classification of state [s1:s1] consensus particles, K = 9, class008 (Scheme 2)	wide rotatory axis angle, subunit j not resolved
**Sub-state [s1:s1g]**	EMD-11454	13.8	〃	3,414	Classification of state [s1:s1] consensus particles, K = 9, class009 (Scheme 2)	narrow rotatory axis angle, subunit j C-terminus resolved
**Sub-state [s1:s2a]**	EMD-11460	17.5	unsharpened	1,069	Classification of state [s1:s2] consensus particles, K = 9, class002 (Scheme 2)	narrow rotatory axis angle, subunit j C-terminus resolved
**Sub-state [s1:s2b]**	EMD-11461	16.9	〃	1,559	Classification of state [s1:s2] consensus particles, K = 9, class003 (Scheme 2)	intermediate rotatory axis angle, subunit j C-terminus resolved
**Sub-state [s1:s2c]**	EMD-11462	16.9	〃	1,532	Classification of state [s1:s2] consensus particles, K = 9, class004 (Scheme 2)	narrow rotatory axis angle, subunit j C-terminus resolved
**Sub-state [s1:s2d]**	EMD-11463	16.9	〃	1,665	Classification of state [s1:s2] consensus particles, K = 9, class005 (Scheme 2)	narrow rotatory axis angle, subunit j C-terminus resolved
**Sub-state [s1:s2e]**	EMD-11464	18.7	〃	977	Classification of state [s1:s2] consensus particles, K = 9, class006 (Scheme 2)	intermediate rotatory axis angle, subunit j C-terminus resolved, partial occupancy

**Sub-state [s1:s2f]**	EMD-11465	15.9	〃	2,357	Classification of state [s1:s2] consensus particles, K = 9, class008 (Scheme 2)	wide rotatory axis angle, subunit j not resolved
**Sub-state [s1:s2g]**	EMD-11466	16.9	〃	1,535	Classification of state [s1:s2] consensus particles, K = 9, class009 (Scheme 2)	intermediate rotatory axis angle, subunit j C-terminus resolved
**Sub-state [s1:s3a]**	EMD-11472	15.9	unsharpened	2,110	Classification of state [s1:s3] consensus particles, K = 9, class001 (Scheme 2)	intermediate rotatory axis angle, subunit j C-terminus resolved
**Sub-state [s1:s3b]**	EMD-11473	18.7	〃	949	Classification of state [s1:s3] consensus particles, K = 9, class002 (Scheme 2)	intermediate rotatory axis angle, subunit j C-terminus resolved not resolved
**Sub-state [s1:s3c]**	EMD-11474	18.7	〃	1,054	Classification of state [s1:s3] consensus particles, K = 9, class003 (Scheme 2)	intermediate rotatory axis angle, subunit j C-terminus resolved, partial occupancy
**Sub-state [s1:s3d]**	EMD-11475	15.9	〃	1,797	Classification of state [s1:s3] consensus particles, K = 9, class005 (Scheme 2)	intermediate rotatory axis angle, subunit j C-terminus resolved
**Sub-state [s1:s3e]**	EMD-11476	20.2	〃	776	Classification of state [s1:s3] consensus particles, K = 9, class006 (Scheme 2)	intermediate rotatory axis angle, subunit j C-terminus resolved not resolved
**Sub-state [s1:s3f]**	EMD-11477	15.4	〃	2,726	Classification of state [s1:s3] consensus particles, K = 9, class007 (Scheme 2)	narrow rotatory axis angle, subunit j C-terminus resolved
**Sub-state [s1:s3g]**	EMD-11479	18.1	〃	1,121	Classification of state [s1:s3] consensus particles, K = 9, class008 (Scheme 2)	wide rotatory axis angle, subunit j not resolved

**Sub-state [s1:s3h]**	EMD-11480	16.9	〃	1,499	Classification of state [s1:s3] consensus particles, K = 9, class009 (Scheme 2)	wide rotatory axis angle, subunit j not resolved
**Sub-state [s2:s1a]**	EMD-11484	16.4	unsharpened	1,846	Classification of state [s2:s1] consensus particles, K = 9, class001 (Scheme 2)	wide rotatory axis angle, subunit j not resolved
**Sub-state [s2:s1b]**	EMD-11485	15.4	〃	2,588	Classification of state [s2:s1] consensus particles, K = 9, class004 (Scheme 2)	narrow rotatory axis angle, subunit j C-terminus resolved
**Sub-state [s2:s1c]**	EMD-11486	17.5	〃	1,334	Classification of state [s2:s1] consensus particles, K = 9, class007 (Scheme 2)	intermediate rotatory axis angle, subunit j C-terminus resolved
**Sub-state [s2:s1d]**	EMD-11487	14.9	〃	2,106	Classification of state [s2:s1] consensus particles, K = 9, class008 (Scheme 2)	narrow rotatory axis angle, subunit j C-terminus resolved
**Sub-state [s2:s2a]**	EMD-11499	17.5	unsharpened	1,258	Classification of state [s2:s2] consensus particles, K = 9, class002 (Scheme 2)	wide rotatory axis angle, subunit j partially resolved
**Sub-state [s2:s2b]**	EMD-11500	17.5	〃	1,459	Classification of state [s2:s2] consensus particles, K = 9, class003 (Scheme 2)	wide rotatory axis angle, subunit j not resolved
**Sub-state [s2:s2c]**	EMD-11501	12.8	〃	3,690	Classification of state [s2:s2] consensus particles, K = 9, class004 (Scheme 2)	narrow rotatory axis angle, subunit j C-terminus resolved, most homogenous
**Sub-state [s2:s2d]**	EMD-11502	16.4	〃	1,422	Classification of state [s2:s2] consensus particles, K = 9, class005 (Scheme 2)	intermediate rotatory axis angle, subunit j C-terminus resolved, partial occupancy

**Sub-state [s2:s2e]**	EMD-11503	18.1	〃	1,247	Classification of state [s2:s2] consensus particles, K = 9, class007 (Scheme 2)	wide rotatory axis angle, subunit j not resolved
**Sub-state [s2:s2f]**	EMD-11504	14.9	〃	2,255	Classification of state [s2:s2] consensus particles, K = 9, class008 (Scheme 2)	intermediate rotatory axis angle, subunit j C-terminus resolved
**Sub-state [s2:s2g]**	EMD-11505	14.9	〃	2,264	Classification of state [s2:s2] consensus particles, K = 9, class009 (Scheme 2)	intermediate rotatory axis angle, subunit j C-terminus resolved
**Sub-state [s2:s3a]**	EMD-11506	18.1	unsharpened	940	Classification of state [s2:s3] consensus particles, K = 9, class001 (Scheme 2)	intermediate rotatory axis angle, subunit j C-terminus resolved
**Sub-state [s2:s3b]**	EMD-11507	19.4	〃	704	Classification of state [s2:s3] consensus particles, K = 9, class002 (Scheme 2)	intermediate rotatory axis angle, subunit j C-terminus not resolved
**Sub-state [s2:s3c]**	EMD-11508	20.2	〃	740	Classification of state [s2:s3] consensus particles, K = 9, class003 (Scheme 2)	wide rotatory axis angle, subunit j not resolved
**Sub-state [s2:s3d]**	EMD-11509	18.1	〃	1,079	Classification of state [s2:s3] consensus particles, K = 9, class004 (Scheme 2)	narrow rotatory axis angle, subunit j C-terminus resolved
**Sub-state [s2:s3e]**	EMD-11510	20.9	〃	619	Classification of state [s2:s3] consensus particles, K = 9, class006 (Scheme 2)	wide rotatory axis angle, subunit j not resolved
**Sub-state [s2:s3f]**	EMD-11511	18.7	〃	938	Classification of state [s2:s3] consensus particles, K = 9, class007 (Scheme 2)	narrow rotatory axis angle, subunit j C-terminus partially resolved

**Sub-state [s2:s3g]**	EMD-11512	16.4	〃	1,695	Classification of state [s2:s3] consensus particles, K = 9, class008 (Scheme 2)	narrow rotatory axis angle, subunit j C-terminus resolved
**Sub-state [s3:s1a]**	EMD-11527	15.4	unsharpened	2,405	Classification of state [s3:s1] consensus particles, K = 9, class001 (Scheme 2)	narrow rotatory axis angle, subunit j C-terminus resolved
**Sub-state [s3:s1b]**	EMD-11528	18.1	〃	1,044	Classification of state [s3:s1] consensus particles, K = 9, class002 (Scheme 2)	intermediate rotatory axis angle, subunit j C-terminus resolved
**Sub-state [s3:s1c]**	EMD-11529	18.1	〃	1,056	Classification of state [s3:s1] consensus particles, K = 9, class003 (Scheme 2)	intermediate rotatory axis angle, subunit j C-terminus partially resolved
**Sub-state [s3:s1d]**	EMD-11530	18.7	〃	867	Classification of state [s3:s1] consensus particles, K = 9, class004 (Scheme 2)	wide rotatory axis angle, subunit j not resolved
**Sub-state [s3:s1e]**	EMD-11531	19.4	〃	874	Classification of state [s3:s1] consensus particles, K = 9, class005 (Scheme 2)	wide rotatory axis angle, subunit j not resolved
**Sub-state [s3:s1f]**	EMD-11532	18.7	〃	927	Classification of state [s3:s1] consensus particles, K = 9, class006 (Scheme 2)	intermediate rotatory axis angle, subunit j C-terminus partially resolved
**Sub-state [s3:s1g]**	EMD-11533	17.5	〃	1,266	Classification of state [s3:s1] consensus particles, K = 9, class008 (Scheme 2)	wide rotatory axis angle, subunit j not resolved
**Sub-state [s3:s1h]**	EMD-11534	17.5	〃	1,254	Classification of state [s3:s1] consensus particles, K = 9, class009 (Scheme 2)	narrow rotatory axis angle, subunit j C-terminus partially resolved

**Sub-state [s3:s2a]**	EMD-11535	16.9	unsharpened	568	Classification of state [s3:s2] consensus particles, K = 9, class001 (Scheme 2)	narrow rotatory axis angle, subunit j C-terminus resolved
**Sub-state [s3:s2b]**	EMD-11536	20.2	〃	1,175	Classification of state [s3:s2] consensus particles, K = 9, class002 (Scheme 2)	intermediate rotatory axis angle, subunit j C-terminus partially resolved
**Sub-state [s3:s2c]**	EMD-11537	18.1	〃	619	Classification of state [s3:s2] consensus particles, K = 9, class003 (Scheme 2)	intermediate rotatory axis angle, subunit j C-terminus resolved
**Sub-state [s3:s2d]**	EMD-11538	20.9	〃	1,003	Classification of state [s3:s2] consensus particles, K = 9, class004 (Scheme 2)	narrow rotatory axis angle, subunit j C-terminus partially resolved
**Sub-state [s3:s2e]**	EMD-11539	18.7	〃	584	Classification of state [s3:s2] consensus particles, K = 9, class005 (Scheme 2)	wide rotatory axis angle, subunit j not resolved
**Sub-state [s3:s2f]**	EMD-11540	20.9	〃	435	Classification of state [s3:s2] consensus particles, K = 9, class006 (Scheme 2)	wide rotatory axis angle, subunit j not resolved
**Sub-state [s3:s2g]**	EMD-11541	20.9	〃	347	Classification of state [s3:s2] consensus particles, K = 9, class008 (Scheme 2)	intermediate rotatory axis angle, subunit j C-terminus partially resolved
**Sub-state [s3:s3a]**	EMD-11542	19.4	unsharpened	818	Classification of state [s3:s3] consensus particles, K = 9, class001 (Scheme 2)	wide rotatory axis angle, subunit j not resolved
**Sub-state [s3:s3b]**	EMD-11543	20.9	〃	588	Classification of state [s3:s3] consensus particles, K = 9, class005 (Scheme 2)	intermediate rotatory axis angle, subunit j C-terminus not resolved

**Sub-state [s3:s3c]**	EMD-11544	17.5	〃	1,179	Classification of state [s3:s3] consensus particles, K = 9, class006 (Scheme 2)	narrow rotatory axis angle, subunit j C-terminus resolved
**Sub-state [s3:s3d]**	EMD-11545	23.8	〃	435	Classification of state [s3:s3] consensus particles, K = 9, class007 (Scheme 2)	wide rotatory axis angle, subunit j not resolved
**Sub-state [s3:s3e]**	EMD-11546	21.8	〃	587	Classification of state [s3:s3] consensus particles, K = 9, class008 (Scheme 2)	intermediate rotatory axis angle, subunit j C-terminus not resolved

[1] The maps were sharpened with an *ad hoc* user defined B-factor in lieu of calculating a value from the half-map data.

[2] Without map sharpening.

Table S3. Summary of the structural models of bovine subunits g, j, and k

Subunit	No. of Residues	Residues modelled
g	102	20-98
j	60	2-49
k	57	12-47

Movie legends

Movie S1 (separate file). Structural heterogeneity in dimeric bovine ATP synthase associated with catalysis. The 13 sec movie demonstrates the rocking motion of the catalytic domains as they step through the three main catalytic states and the corresponding contortions of the PS as it responds to the torque of rotation, and also the subtle changes in the spatial relationship of each monomer to the other as the enzyme cycles through catalytic states and how this changes the angle of the rotatory axis. In Movie S2, these subtle changes can be seen also by tracking the movement of subunit e (khaki) and subunit g (forest green). The range of observed angles between rotatory axes in the consensus reconstructions was *ca.* 76° to 86°. In the membrane, it is likely that these movements will be damped by the lateral pressure of the bilayer and by interactions with adjacent dimers. This damping would result in larger apparent motions of the PS and catalytic domain (which can be demonstrated by aligning monomers of state 1, state 2 and state 3 via their a-subunits, that is, by fixing the relative motion of the membrane domains). Conversely, this fluidity in the membrane domain may provide a mechanism to dampen motions caused by asymmetric rotation by reducing the net displacement of the catalytic domain.

Movie S2 (separate file). The rotary cycle during synthesis and hydrolysis. In the synthetic direction, reconstructions appear in the order state [s3:s1] > [s1:s2] > [s2:s3], and the rotor turns anticlockwise as viewed from the mitochondrial matrix; during hydrolysis with clockwise rotation, the order is reversed. This 21 sec movie complements Movie S1 by identifying the subunits of the enzyme and by displaying the reconstructions in the physiological order of catalysis. From 00:00-00:06 sec, the reconstructions are shown slowly in order, from 00:06 sec to 00:10 sec, the enzyme is undergoing synthesis, and from 00:10 sec to 00:14 sec hydrolysis. Then the sequence from 00:06 sec to 00:14 sec is repeated. Although the enzyme is inhibited by IF_1, it is possible to construct the rotary mechanism because all three states of the catalytic β-subunits, β_E, β_{DP} and β_{TP}, are present in the structure. In the intact monomer, the three-fold rotational symmetry of the mechanism is broken by the association of the PS, and the three IF_1 inhibited rotational states represent the full catalytic cycle in any of the three catalytic sites. As the maps or models appear in sequence, each catalytic site cycles through the β_E, β_{DP} and β_{TP} states during synthesis, and the reverse sense during hydrolysis. As shown in the movie, the dimeric enzyme contorts significantly during the independent rotary cycles of each monomer. The α, β, γ, δ, ε, OSCP and F_6 subunits of the membrane extrinsic domain are dull red, golden yellow, purple-blue, green, dark purple, teal and magenta, respectively. The inhibitor protein, IF_1, (grey) protrudes from the α_{DP}-β_{DP} interface in various positions. The membrane domain subunits a, A6L, b, e, f, g, j and k are cornflower blue, brick red, light pink, khaki, straw yellow, forest green, sea-foam green and dark pink, respectively. The phosphates of the lipid headgroups are bright red. The c_8-ring and remaining unmodelled density of the micelle are dark grey and light grey, respectively.

Movie S3 (separate file). Pivoting of the membrane domains of adjacent monomers of bovine ATP synthase about the matrix contact between j-subunits during catalysis. The 53 sec movie, starts with the consensus reconstruction of the ATP synthase dimer in state [s1:s2] (grey) followed by a schematic representation of the monomeric models rigid body fitted into the density. Then from 00:05 sec to 00:013 sec, the state [s2:s3] and state [s3:s1] dimers and their atomic models appear in catalytic order during synthesis. From 00:14 sec to 00:30 sec, side and top views of the enzyme highlight the rearrangement of the membrane domains during synthesis. In

particular, there is significant movement in the relative positions of subunits f (straw yellow) and g (forest green), which rotate with the rest of the membrane domain during the pivoting motion. Finally, transitions between pairs of catalytic states are alternated to accentuate each component of the total movement. From 00:30 sec to 00:37 sec are shown the transition from state [s3:s1] to state [s1:s2]; followed by the transition from state [s1:s2] to state [s2:s3] from 00:38 sec to 00:45 sec, and the transition from state [s2:s3] to state [s3:s1] from 00:45 sec to 00:53 sec; in the top view, the changes at the interface between the two j-subunits can be seen, about which the monomers pivot during the catalytic cycle. For colors of subunits and other details, see the legend to Fig. 1.

Movie S4 (separate file). The fluidity, independent of catalysis, of the monomer:monomer interface at rotatory axis angles less than or equal to 90°. The 56 sec movie depicts a transition of the state [s2:s2] sub-states from sub-state [s2:s2c] > [s2:s2f] > [s2:s2g] > [s2:s2c]. The sub-states were derived by classification of ATP synthase dimer particles in rotational state [s2:s2]. Thus, the pivoting is independent of both the action of catalysis and the associated motions described in Movies 1 and 2, although the movement mode is similar. The transitions between these sub-states demonstrate the pivoting motion about the subunit j:subunit j interface in the centre of the membrane domain, with the accompanying rearrangement of subunits e, f, g and k. In the first part of the movie (00:00 sec to 00.21 sec) a side view, in the plane of the membrane, is presented, and in the second part, from 00:22 sec to 00:40 sec, the same sub-state transitions are viewed from the mitochondrial matrix. From 00:40 sec to 00:56 sec, the view is from the IMS showing the C-terminal contact point of subunit j protruding into the IMS. Several sub-states displaying this feature were found in all consensus dimer particle sub-sets and, when combined, they represent the most abundant structural conformers in the dataset with 67.6 % of particles belonging to classes in which the reconstruction displays evidence of contact between the C-terminal regions of j-subunits. In general, the foot-print of the membrane domain of these sub-states is wider, and the angle between the central axes of the F_1-c_8 domains is narrower with a correspondingly shorter distance between F_1 domains. The membrane domain subunits a, A6L, b, e, f, g, j and k are cornflower blue, brick red, light pink, khaki, straw yellow, forest green, sea-foam green and dark pink, respectively. The c_8-ring and remaining unmodelled density of the micelle are dark grey and light grey, respectively.

Movie S5 (separate file). Trajectory, independent of catalysis, towards the formation of a wide angle between the central axes of the F_1-c_8 domains in a dimer of ATP synthase. The widening arises from a sliding and twisting of the membrane domains along the monomer-monomer interface surface provided by subunits a and j and occurs independently from the action of catalysis. The 36 sec movie displays transitions between several sub-states with more obtuse rotatory axis angles than those in the consensus reconstructions (shown in Movies S1, S2 and S3) and in the other sub-states of the state [s2:s2] dimer (see Movie S4). From 00:00 sec to 00:06 sec, the density of the dimer reconstructions is colored to match the subunit colors of the composite atomic models fitted within them (see Fig. 1). From 00:07 sec to 00:15 sec, the densities are interpolated in the following order; sub-states [s2:s2c], [s2:s2a], [s2:s2e], [s2:s2b], [s2:s 2e], then [s2:s2c] and so forth. This sequence is repeated in a view from the matrix and IMS sides of the complex from 00:19 sec 00:24 sec and 00:31 sec to 00:36 sec, respectively. The dramatic changes in the angle of the rotatory axes and the substantial rearrangement of the dimer interface thins the foot-print of the membrane domain significantly (as viewed from above or below the complex). This thinning affects how dimers of dimers and higher oligomers might form, as in this configuration subunit k is positioned closer to subunit g in an adjacent dimer. Also, the IMS protrusion of subunit j is absent, possibly because either a contact between the C-terminal regions of the subunit has been broken, or because of the lower resolution of these reconstructions. Other possible explanations are that this wide angle conformation results from the loss of one or all of the following: subunit j, CDL1, or unresolved lipids in the wedge. It is also possible that the extended structure of the C-terminal region of subunit j remains in contact, but was not resolved. This is possible, if not probable, as the distance between the last modelled residues in the subunit j protomers is *ca.* 29-38 Å at its furthest in sub-state [s2:s2b] and the C-terminal 11 residues of subunit j were not built. If the unresolved

region comprises an extended and/or a partially α-helical structure, 22 residues would be sufficient to span the distance between their C-termini.

Movie S6 (separate file). Detailed view of the interface rearrangement, independent of catalysis, arising in the trajectory towards the wide angle dimeric ATP synthase. A top view of the trajectory in Movie S5 is shown. Subunits a and j are cornflower blue and sea-foam green, respectively, and are shown as a solvent excluded surface. The 41 sec movie shows the sliding and twisting of each monomer along the monomer-monomer interface that leads to the widest of the rotatory axis angles, and the narrowest membrane domain footprint. The final part of the movie (from 31 sec) depicts the same sequence of events in a side view of the dimeric complex, shown as a solvent excluded molecular surface. This final part demonstrates how the changes in the dimer interface depicted in the first portion of the movie relate to changes of the rotatory axis between the monomers.

Movie S7 (separate file). Catalytic and structural heterogeneity in purified dimeric bovine ATP synthase. This 38 sec movie shows all the motions observed between monomers accompanying changes in rotational state, and flexions of monomers in fixed rotational states, and combinations of both. The same sequence of reconstructions appear three times at increasing speeds to demonstrate the highly dynamic nature of the assembly. From 00:00 sec to 00:18 sec, in a side view of the dimer, the reconstructions are changed at a rate of *ca.* 3 sec^{-1}. In the second part from 00:18 sec to 00:26 sec the rate is *ca.* 9 sec^{-1}. In the final segment from 27 sec to 38 sec, the rate is *ca.* 24 sec^{-1} and the complexes are viewed from the matrix. All combinations of rotational state and the extents of the relative motions of the monomers are represented.

SI References

1. T.E. Spikes, M.G. Montgomery, J.E. Walker, Structure of the dimeric ATP synthase from bovine mitochondria. *Proc. Natl. Acad. Sci. U. S. A.* (2020).
2. S.H. Scheres, RELION: implementation of a Bayesian approach to cryo-EM structure determination. *J. Struct. Biol.* **180**, 519-530 (2012).
3. S.H. Scheres, A Bayesian view on cryo-EM structure determination. *J. Mol. Biol.* **415**, 406-418 (2012).
4. R. Fernandez-Leiro, S.H.W. Scheres, A pipeline approach to single-particle processing in RELION. *Acta Crystallogr D Struct Biol* **73**, 496-502 (2017).
5. J. Zivanov *et al.*, New tools for automated high-resolution cryo-EM structure determination in RELION-3. *Elife* **7**, (2018).
6. J. Zivanov, T. Nakane, S.H.W. Scheres, Estimation of high-order aberrations and anisotropic magnification from cryo-EM data sets in RELION-3.1. *IUCrJ* **7**, 253-267 (2020).
7. P. Emsley, B. Lohkamp, W.G. Scott, K. Cowtan, Features and development of Coot. *Acta Crystallogr D Biol Crystallogr* **66**, 486-501 (2010).
8. P.V. Afonine *et al.*, Real-space refinement in PHENIX for cryo-EM and crystallography. *Acta Crystallogr D Struct Biol* **74**, 531-544 (2018).
9. D. Liebschner *et al.*, Macromolecular structure determination using X-rays, neutrons and electrons: recent developments in Phenix. *Acta Crystallogr D Struct Biol* **75**, 861-877 (2019).
10. T.D. Goddard *et al.*, UCSF ChimeraX: Meeting modern challenges in visualization and analysis. *Protein Sci* **27**, 14-25 (2018).
11. J. Gu *et al.*, Cryo-EM structure of the mammalian ATP synthase tetramer bound with inhibitory protein IF_1. *Science* **364**, 1068-1075 (2019).
12. F. Madeira *et al.*, The EMBL-EBI search and sequence analysis tools APIs in 2019. *Nucleic Acids Res.* **47**, W636-W641 (2019).
13. D.T. Jones, Protein secondary structure prediction based on position-specific scoring matrices 1 1Edited by G. Von Heijne. *J. Mol. Biol.* **292**, 195-202 (1999).
14. D.W.A. Buchan, D.T. Jones, The PSIPRED Protein Analysis Workbench: 20 years on. *Nucleic Acids Res.* **47**, W402-W407 (2019).
15. D.W. Buchan, F. Minneci, T.C. Nugent, K. Bryson, D.T. Jones, Scalable web services for the PSIPRED Protein Analysis Workbench. *Nucleic Acids Res.* **41**, W349-57 (2013).
